# Alterations of Placental Sodium in Preeclampsia: Trophoblast Responses

**DOI:** 10.1161/HYPERTENSIONAHA.124.23001

**Published:** 2024-07-05

**Authors:** Hiten D. Mistry, Rahel Klossner, Paula J. Scaife, Nicole Eisele, Lesia O. Kurlak, Sampada Kallol, Christiane Albrecht, Carine Gennari-Moser, Louise V. Briggs, Fiona Broughton Pipkin, Markus G. Mohaupt

**Affiliations:** Department of Women and Children’s Health, School of Life Course and Population Health Sciences, King’s College London, United Kingdom (H.D.M.).; Teaching Hospital Internal Medicine, Lindenhofgruppe, Switzerland (R.K., M.G.M.).; Department of Nephrology and Hypertension (R.K., N.E., C.G.-M., M.G.M.), University of Bern, Switzerland.; Department for BioMedical Research (R.K., N.E., C.G.-M., M.G.M.), University of Bern, Switzerland.; Clinical, Metabolic and Molecular Physiology (P.J.S.), University of Nottingham, United Kingdom.; Stroke Trials Unit (School of Medicine) (L.O.K.), University of Nottingham, United Kingdom.; Advanced Material Research Group, Faculty of Engineering (L.V.B.), University of Nottingham, United Kingdom.; Institute for Biochemistry and Molecular Medicine, University of Bern, Switzerland (S.K., C.A.).; Department of Obstetrics, City Hospital Nottingham, United Kingdom (F.B.P.).

**Keywords:** adenosine triphosphatases, biopsy, preeclampsia, sodium, trophoblasts

## Abstract

**BACKGROUND:**

Evidence suggests that increasing salt intake in pregnancy lowers blood pressure, protecting against preeclampsia. We hypothesized that sodium (Na^+^) evokes beneficial placental signals that are disrupted in preeclampsia.

**METHODS:**

Blood and urine were collected from nonpregnant women of reproductive age (n=26) and pregnant women with (n=50) and without (n=55) preeclampsia, along with placental biopsies. Human trophoblast cell lines and primary human trophoblasts were cultured with varying Na^+^ concentrations.

**RESULTS:**

Women with preeclampsia had reduced placental and urinary Na^+^ concentrations, yet increased urinary angiotensinogen and reduced active renin, aldosterone concentrations, and osmotic response signal TonEBP (tonicity-responsive enhancer binding protein) expression. In trophoblast cell cultures, TonEBP was consistently increased upon augmented Na^+^ exposure. Mechanistically, inhibiting Na^+^/K^+^-ATPase or adding mannitol evoked the TonEBP response, whereas inhibition of cytoskeletal signaling abolished it.

**CONCLUSIONS:**

Enhanced Na^+^ availability induced osmotic gradient-dependent cytoskeletal signals in trophoblasts, resulting in proangiogenic responses. As placental salt availability is compromised in preeclampsia, adverse systemic responses are thus conceivable.

**NOVELTY AND RELEVANCE**

**What Is New?**

- These findings highlight the placenta’s role as a previously unrecognized sensor of salt levels, complementing the role of the kidneys.

**What Is Relevant?**

- The breakdown in the mechanism responsible for retaining salt indicates potential abnormalities in Na^+^-related signals within the placenta.

**Clinical/Pathophysiological Implications?**

- Maintaining an appropriate level of Na^+^ exposure in the placenta, such as through increased dietary salt intake, could be explored as a potential early preventive or therapeutic approach for preeclampsia.

Preeclampsia is a leading cause of maternal and fetal morbidity and mortality worldwide.^1^ It is associated with lifelong consequences for both mother and her child, including increased risks of cardiovascular and metabolic diseases.^2^

During pregnancy, women retain a total of 500 to 900 mmol sodium (Na^+^), while the plasma volume increases by 30% to 50%.^3^ Counterintuitively, this does not translate into a rise in blood pressure,^4^ due to vasodilatation related to angiogenic factors.^5^ In contrast, lower plasma volume in preeclampsia is associated with hypertension, suggesting an inverse linkage between blood pressure and plasma volume.^6^ This is further supported by the vain attempts to prevent preeclampsia by lowering dietary Na^+^ intake or increasing Na^+^ excretion via diuretics.^7,8^

Renal Na^+^ retention raising the plasma volume is considered secondary to a renin-angiotensin system dependent, as well as a renin-angiotensin system independent VEGF vascular endothelial growth factor)-augmented aldosterone synthesis, previously described by our group.^9,10^ This is further amplified by a direct action of angiotensin II on the proximal tubule and by an activating posttranslational cleavage of subunits of the epithelial Na^+^ channel in the cortical collecting duct, as we previously reported.^11,12^

The relevance of appropriate Na^+^ availability is based on animal models and human diseases with aldosterone synthase deficiency. These data suggest that Na^+^ supplementation can restore an aldosterone-replete phenotype.^13,14^ Furthermore, increasing salt intake in pregnancy lowered blood pressure and protected against preeclampsia^14,15^; similar data by our group is observed in the first trimester of human pregnancy^12^ and in pregnant animal models.^13,16,17^ However, both, most susceptible individuals and potential effector mechanisms, are yet insufficiently defined.

Large amounts of Na^+^ are stored in the skin interstitium, leading to functional consequences,^5,18,19^ although it is appreciated that Na^+^ is also stored in other tissues (eg, muscle). TonEBP (Tonicity-responsive enhancer binding protein), also known as the NFAT5 (nuclear factor of activated T-cells 5), is a signal transcription factor activated upon osmotic changes. Na^+^ accumulation is sensed by dendritic cells (DCs), activating TonEBP, which then stimulates vascular endothelial growth factor-C (VEGF-C).^20,21^ Disruption of this TonEBP-VEGF-C axis in rats increased blood pressure.^5^ TonEBP is abundantly expressed in the human term placenta in physiological pregnancies,^22^ yet its function is unknown.

TonEBP is required for the maturation and function of DCs and is involved in the pathogenesis of autoimmune diseases and inflammation.^23^ The placenta exhibits a DC-like phenotype,^24^ and the breakdown of its immune tolerance may contribute to preeclampsia.^25^

Given the functional observations and the DC-like phenotype of trophoblasts, we hypothesized that Na^+^ evokes placental/trophoblastic signals, which are beneficial for a healthy pregnancy and that maintained placental Na^+^ availability is essential to pregnancy.

Thus, we aimed to measure placental Na^+^ availability and to identify Na^+^-induced changes in trophoblast signaling, as modeled by exposing trophoblast cell lines and primary human trophoblasts to different Na^+^ concentrations.

## METHODS

### Data Availability

The data that support the findings of this study are available from the corresponding author upon reasonable request.

Details of participant, recruitment, sample collection, and methodology used are provided in the Supplemental Material. Briefly, urinary and placental Na^+^ concentrations were measured using inductively coupled mass spectrometry.^26^ Blood and urine renin-angiotensin system concentrations were measured using ELISA kits. Urinary tetrahydroaldosterone was measured using gas chromatography–mass spectrometry. All urinary concentrations were corrected for creatinine concentrations. Cell culture experiments were completed on isolated primary trophoblast cells, BeWo (CCL94), JEG-3 (HTB36), and HTR-8/SVneo cell lines.^27^ Expression of mRNA and protein was measured using quantitative real-time PCR (RT-PCR) and Western blot, respectively.

### Statistical Analysis

All graphs and data are presented as mean±SD or median interquartile range (IQR), as appropriate for the data distribution. Data from human placentae were analyzed by Mann-Whitney *U* test. Data from the cell lines were analyzed by repeated measures 2-way ANOVA and Tukey multiple comparisons test to compare ≥3 means, and Sidak multiple comparisons test to compare only 2 means. Data from primary cytotrophoblasts were analyzed by repeated measures 1-way ANOVA and Tukey multiple comparisons test. The null hypothesis was rejected at *P*<0.05. All statistical analyses were performed using SPSS (version 26; IBM) and GraphPad Prism (version 8; GraphPad Software).

## RESULTS

### Clinical Samples

The Table summarizes the demographic data and pregnancy outcome of the women recruited into this study. Throughout, controls refer to normotensive pregnant women.

**Table.**
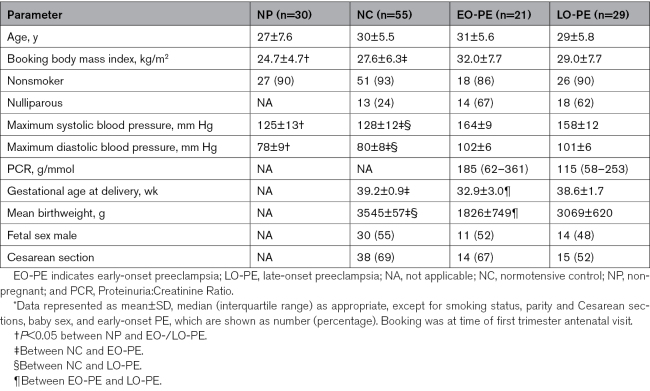
Clinical and Obstetric Data of Subject Groups^*^

### Urinary Na^+^ Concentrations

**Figure 1.**
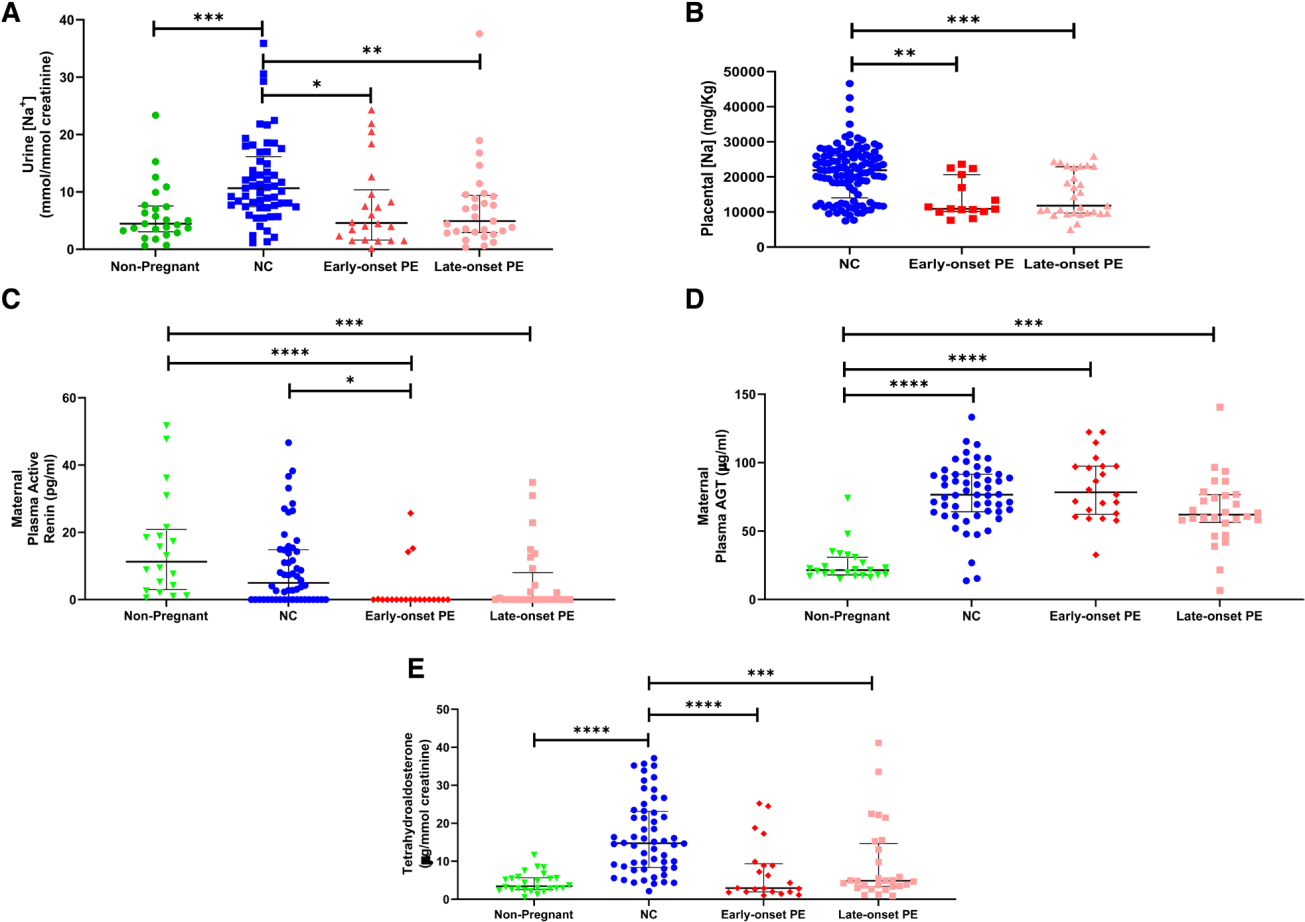
**Urinary and placental sodium and renin-angiotensin-aldosterone concentrations in nonpregnant women; normotensive control (NC) and preeclampsia (PE).** Data are shown separately for early (diagnosis ≤34 weeks) and late (diagnosis >34 weeks) onset PE. **A**, Urinary sodium corrected for creatinine (nonpregnant, n=26; NC, n=55; early-onset PE, n=21; late-onset PE, n=29); (**B**) human placental sodium (NC, n=55; early-onset PE, n=15; late-onset PE, n=28); (**C**) maternal plasma active renin (nonpregnant, n=26; NC, n=55; early-onset PE, n=21; late-onset PE, n=29); (**D**) maternal plasma (angiotensinogen; nonpregnant, n=26; NC, n=55; early-onset PE, n=21; late-onset PE, n=29); and (**E**) urine tetrahydroaldosterone/creatinine (nonpregnant, n=26; NC, n=55; early-onset PE, n=21; late-onset PE, n=29). Data presented as median (interquartile range)], *P<0.05, **P<0.01, ***P<0.001, ****P<0.0001.

Urinary Na^+^ concentrations differed between all groups (*P*<0.05) and were higher in normotensive pregnant controls compared with nonpregnant women (*P*<0.01; Figure 1A). All women with preeclampsia had urinary Na^+^ concentrations lower than normotensive pregnant controls (*P*<0.0001). When further subgrouped, urinary Na^+^ concentrations in early- and late-onset preeclampsia were lower than normotensive controls (*P*<0.05 for both; Figure 1A).

### Lower Na^+^ Content in Term Human Placentae From Women With Preeclampsia

Differences in placental Na^+^ concentrations were observed between all groups (*P*<0.05). Placentae from all women with preeclampsia had markedly lower Na^+^ content (*P*<0.05 for all; Figure 1B) compared with those of normotensive women.

### Blood and Urine Renin-Angiotensin System Concentrations

Plasma active renin concentrations were different between groups (*P*<0.05), with the highest in nonpregnant women (*P*<0.05; Figure 1C), when compared with normotensive pregnancy; women with preeclampsia had even lower plasma active renin concentrations (*P*<0.0001), a difference that was maintained when subgrouped by early-onset preeclampsia only (Figure 1C).

Maternal plasma AGT (angiotensinogen) concentrations differed between groups (*P*<0.05) with all pregnancy groups having higher concentrations than nonpregnant women (*P*<0.0001 for all; Figure 1D).

Tetrahydroaldosterone:creatinine ratios were different between groups (*P*<0.05), with an increase in normotensive controls when compared with the nonpregnant group (*P*<0.001; Figure 1E). Aldosterone excretion was lower in the preeclampsia group as compared with normotensive controls (*P*<0.0001) and independent of the higher body mass index (BMI) in women with preeclampsia.

### Cell Viability

To assess the impact of different cell culture treatments on cell viability, we used the MTT (3-(4, 5-dimethylthiazolyl-2)-2, 5-diphenyltetrazolium bromide) assay. More than 70% viability at the end of the experiment was considered acceptable, and this was achieved in all experiments performed, with 2 exceptions: after 24 hours of incubation with 170 mmol/L Na+, only 65% of BeWo cells were viable, and after inhibition of Na+/K+-ATPase by ouabain, only 41% of BeWo cells were viable (Table S4).

### Effect of NaCl on TonEBP, SMIT, VEGF-C, Flt-1, and PlGF mRNA Expression in Human Trophoblast Cell Lines and in Human Primary Term Cytotrophoblasts

**Figure 2.**
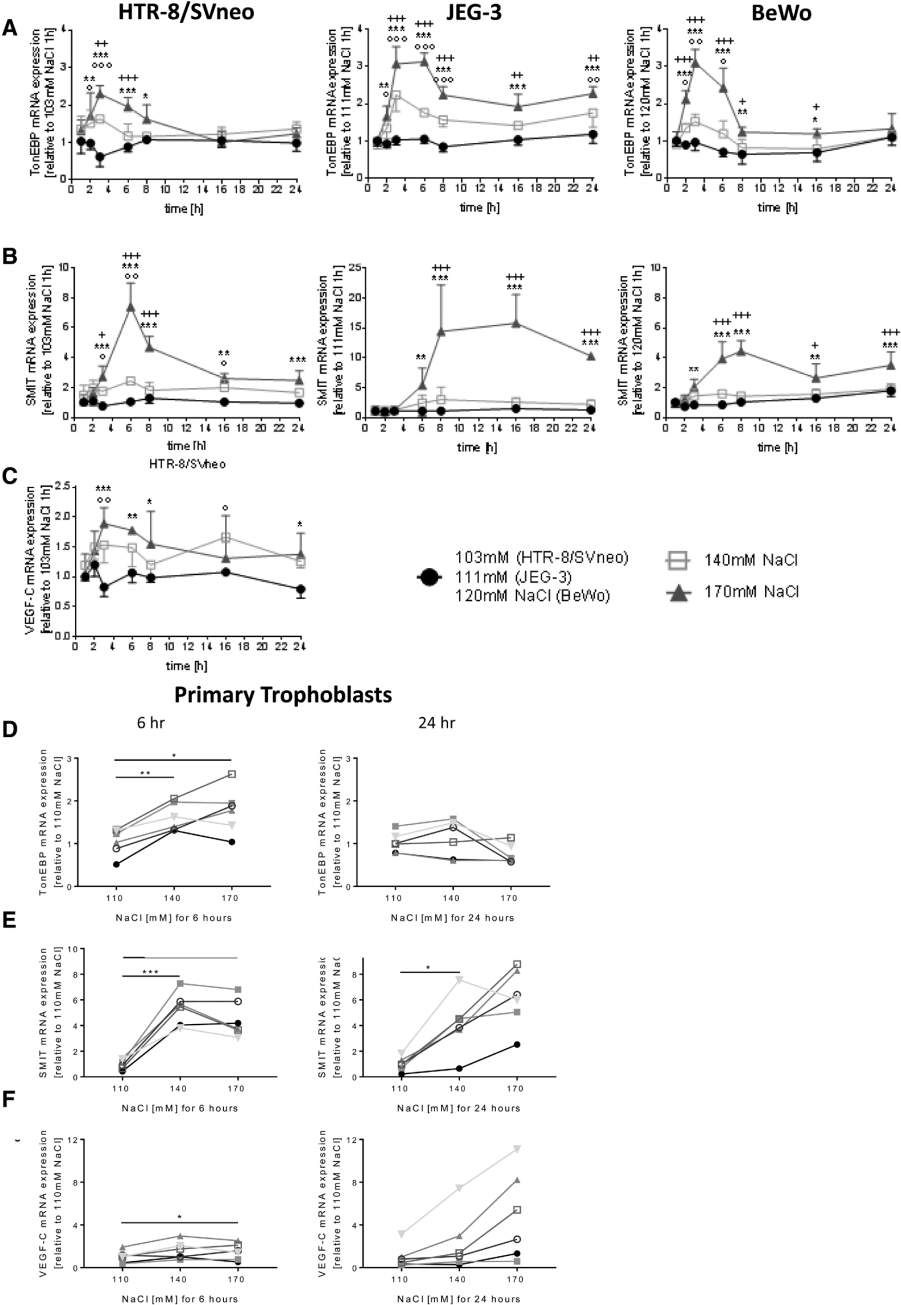
**Effect of different NaCl concentrations on trohophoblast expression of TonEBP (tonicity-responsive enhancer binding protein), SMIT (sodium myo-inositol cotransporter), and VEGF-C (vascular endothelial growth factor-C).** Human trophoblast cell lines (**A–C**), and isolated primary term cytotrophoblasts (**D–F**). The human trophoblast cell lines HTR-8/SVneo (first trimester trophoblasts), JEG-3, and BeWo (trophoblasts, derived from choriocarcinoma cell lines) were incubated with normal (103 mmol/L for HTR-8/SVneo, 111 mmol/L for JEG-3, 120 mmol/L for BeWo) or high NaCl concentrations (140–170 mmol/L) for 1 to 24 hours. The mRNA expression of TonEBP, SMIT, and VEGF-C was measured by quantitative PCR (qPCR). **A** and **B**, High NaCl increased TonEBP and its downstream gene *SMIT* in all cell lines; (**C**) VEGF-C could only be quantified in HTR-8/SVneo and increased with high NaCl. Human primary term cytotrophoblasts were incubated with normal (110 mmol/L) or high NaCl concentrations (140–170 mmol/L) for 6 (**left**) or 24 hours (**right**). **D**, High NaCl increased TonEBP, (**E**) its downstream genes *SMIT* and (**F**) *VEGF-C*. **A** through **C**, Data are presented as mean±SD (n=3 biological replicates). Error bars are shown only in 1 direction for better readability. For some points, the error bars would be shorter than the height of the symbol and are not displayed. **D** through **F**, Each symbol and shade of gray represents a different trophoblast isolation from an individual placenta (n=6). *, ○, or ^+^*P*<0.05; **, ○○, or ^++^*P*<0.01; ***, ○○○, or ^+++^*P*<0.001. * shows significances between 170 mmol/L NaCl and normal NaCl (103 mmol/L for HTR-8/SVneo, 111 mmol/L for JEG-3, 120 mmol/L for BeWo). ○ shows significances between 140 mmol/L NaCl and normal NaCl. ^+^ shows significances between 170 mmol/L and 140 mmol/L NaCl. *P<0.05, **P<0.01, ***P<0.001.

The human trophoblast cell lines HTR-8/SVneo, JEG-3, and BeWo were exposed to different NaCl levels (103–120 mmol/L) or supraphysiological NaCl concentrations (up to 170 mmol/L) for 1 to 24 hours. In all cell lines, NaCl dose dependently increased *TonEBP* mRNA expression as compared with physiological NaCl concentrations, peaking between 3 and 6 hours of exposure (Figure 2A). In HTR-8/SVneo and BeWo cells, *TonEBP* mRNA expression returned to baseline levels after 24 hours, whereas transcripts remained elevated in JEG-3 at 24 hours (Figure 2A). TonEBP protein expression was in line with the mRNA data in all 3 cell lines (Figure S1). *SMIT* (*Sodium myo-inositol cotransporter*) mRNA expression dose dependently increased with rising NaCl concentrations and peaked a few hours later than *TonEBP* expression (Figure 2B). Furthermore, TonEBP and *SMIT* expression also increased in BeWo stimulated with forskolin (data not shown).

BeWo and JEG-3 cells only marginally expressed VEGF-C, a downstream signal of TonEBP (cycling time>35; Table S2). In contrast, HTR-8/SVneo cells highly expressed basal *VEGF-C* levels (cycling time=23), which were upregulated 2-fold upon high NaCl exposure (Figure 2C).

As Flt-1 (vascular endothelial factor receptor 1) is to some extent alternatively spliced, thus leading to soluble forms pathogenically relevant to preeclampsia, we assessed its expression in response to NaCl. High NaCl concentrations initially increased *Flt-1* mRNA expression, with only moderate changes beyond 8 hours in HTR-8/SVneo, whereas the response in JEG-3 cells was at later times, pointing toward a late suppression by high NaCl levels (Figure S2A). Flt-1 mRNA expression was missing in BeWo (Table S2).

Supraphysiological NaCl concentrations stimulated PlGF (placental growth factor) mRNA expression in HTR-8/SVneo and JEG-3 cells. In contrast, BeWo did not respond to increasing NaCl concentrations (Figure S2B).

Similar experiments in 6 independent isolations of primary human term cytotrophoblasts confirmed the results above. High NaCl concentrations stimulated *TonEBP* expression as early as 6 hours with no late response at 24 hours (Figure 2D). The *SMIT* transcript levels were also significantly increased at 6 hours and remained elevated for 24 hours (Figure 2E). Increasing NaCl concentrations stimulated *VEGF-C* mRNA expression after 24 hours in primary human term cytotrophoblasts (Figure 2F).

All third trimester trophoblast samples unambiguously indicated a suppression of Flt-1 transcripts at 24 hours (Figure S2C), a finding which was also present in most isolates for *PlGF* (Figure S2D).

In summary, secondary signals of trophoblast Na^+^ exposure included the upregulation of TonEBP, SMIT, and VEGF-C.

### Mechanism of TonEBP Activation in Response to High NaCl

As it is not yet known which factors play a major role in TonEBP activation in the placenta, we first addressed the guanine nucleotide exchange factor Brx, an important TonEBP regulator in lymphocytes.^28^ Brx knockdown by siRNA did not reduce the expression of *TonEBP* or *SMIT* (Figure S3) upon exposure to increased NaCl levels.

**Figure 3.**
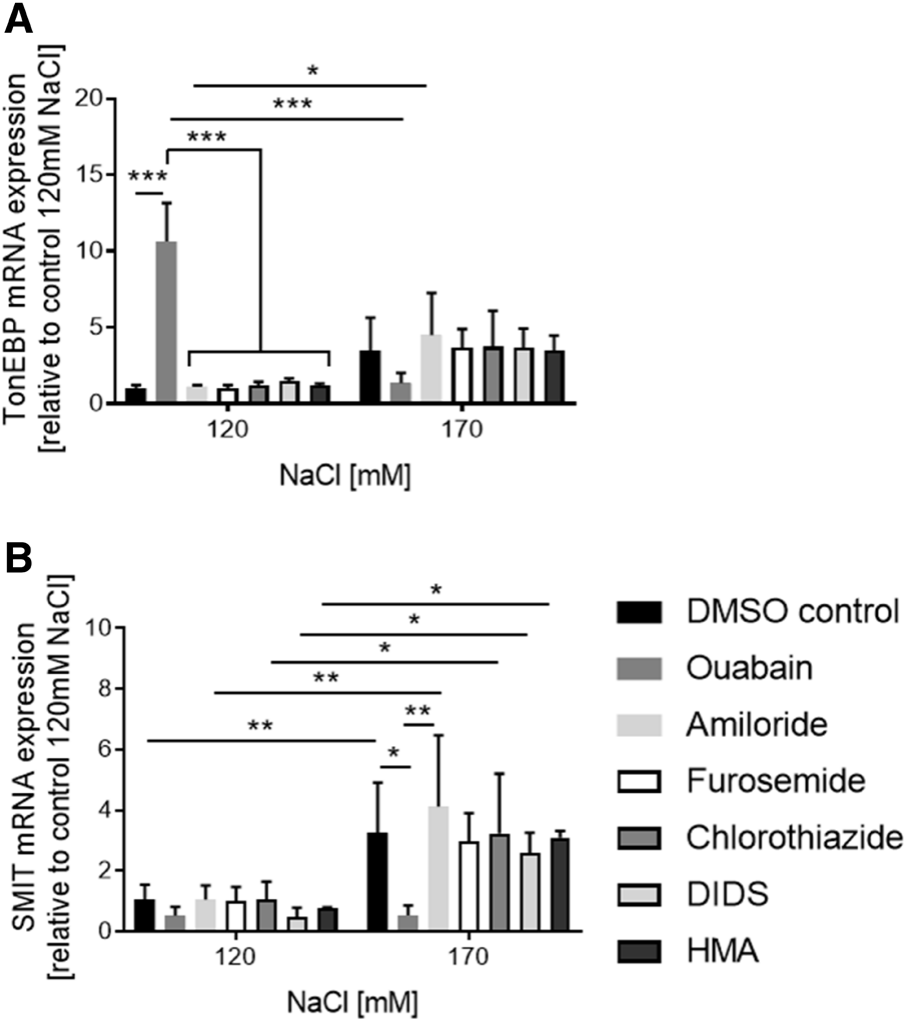
**Mechanism of TonEBP** ( **tonicity-responsive enhancer binding protein) activation in response to high NaCl.** BeWo cells were incubated with inhibitors of NaCl transporters in normal (120 mmol/L) and high NaCl(170 mmol/L) for 6 hours. Na/K-ATPase was inhibited by 10^−4^ M ouabain, epithelial sodium channel by 10^−5^ M amiloride, Na-K-2Cl cotransporter by 10^−4^ M furosemide, NaCl cotransporter by 10^−4^ M chlorothiazide, anion exchanger by 10^−4^ M 4,4′-Diisothiocyano-2,2′-stilbenedisulfonic acid (DIDs), and sodium-proton exchanger by 10^−6^ M 5-(*N*,*N*-hexamethylene)amiloride. The mRNA expression of (**A**) TonEBP and (**B**) SMIT (sodium myo-inositol cotransporter) was measured by quantitative PCR (qPCR). None of the NaCl-influx-transporter inhibitors (amiloride, furosemide, chlorothiazide, 4,4′-diisothiocyano-2,2′-stilbenedisulfonic acid, 5-(*N*,*N*-hexamethylene)amiloride) prevented the TonEBP and SMIT increase at high NaCl. Only inhibition of the **sodium** -efflux (ouabain) via the essential Na/K-ATPase affected the TonEBP and SMIT expression. Data are presented as mean±SD (n=3 biological replicates). *P<0.05, **P<0.01, ***P<0.001.

As numerous intracellular signals are induced by Na^+^ fluxes, the role of several NaCl transporters in TonEBP activation and inhibition in BeWo cells was investigated: Na^+^-influx via the epithelial Na^+^ channel,^29^ the Na^+^-K^+^-2Cl^−^ cotransporter,^30^ the Na^+^-Cl^−^ cotransporter,^31^ Cl^−^ channels,^32^ and the Na^+^-H^+^ exchanger^33^ in medium equilibrated to normal (120 mmol/L) or high NaCl (170 mmol/L). Transcript levels of *TonEBP* and *SMIT*, induced in response to high NaCl, were not affected by the specific Na^+^-influx inhibitors (Figure 3A and 3B). However, blocking Na^+^-efflux via Na^+^/K^+^-ATPase inhibition (ouabain) resulted in enhanced and abolished *TonEBP* responses upon incubation with low and high NaCl levels, respectively (Figure 3A), consistent with altered intracellular osmolality. In low NaCl conditions, the *TonEBP* increase was not translated into an *SMIT* response, despite high TonEBP mRNA expression upon inhibition with ouabain (Figure 3B).

In summary, TonEBP was not regulated by Brx or altered by individual Na^+^ channels, though severe alterations of intracellular Na^+^ availability by interfering with Na^+^/K^+^-ATPase did result in altered TonEBP expression.

**Figure 4.**
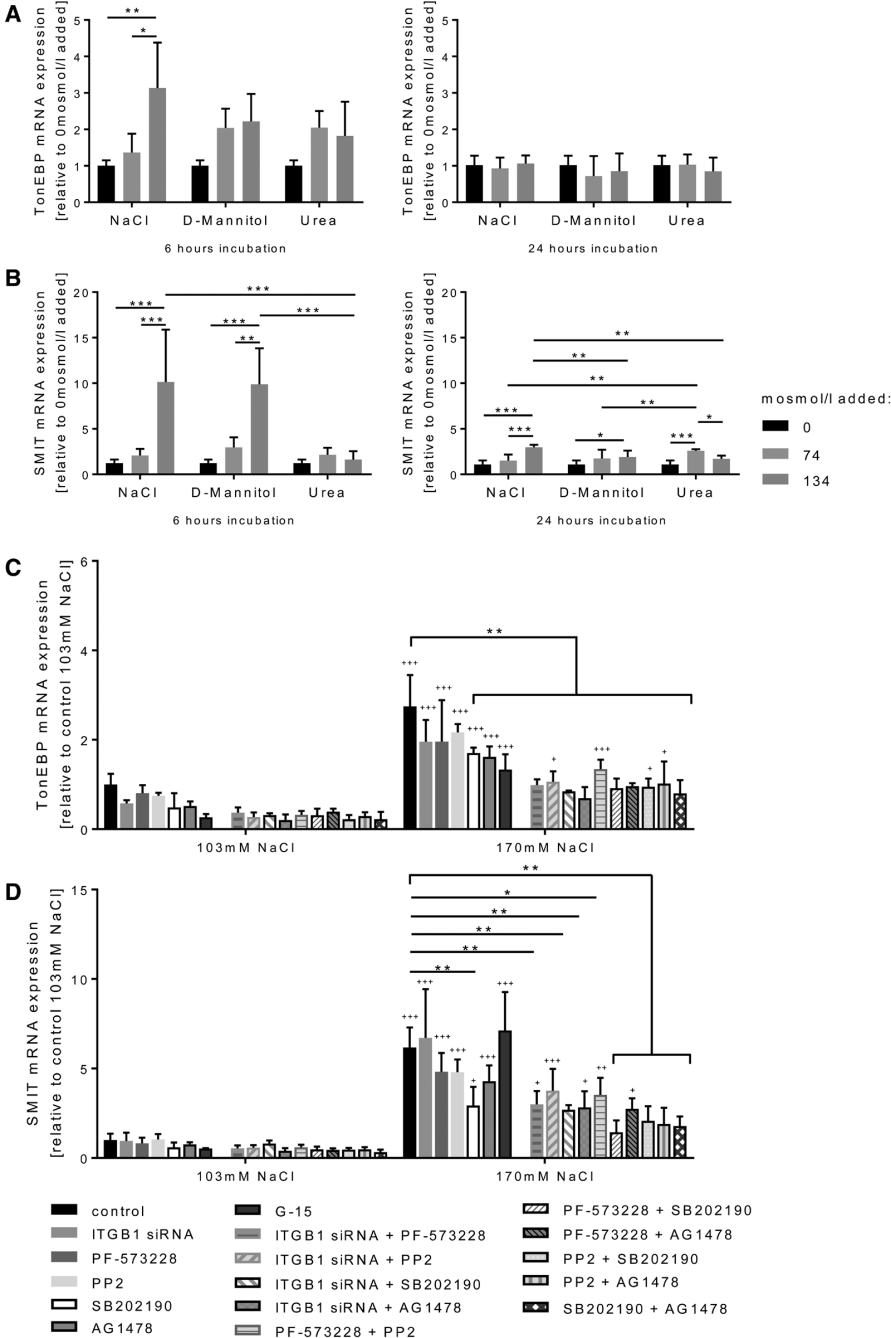
**Mechanism of TonEBP** ( **tonicity-responsive enhancer binding protein) activation in response to high NaCl.** HTR-8/SVneo cells were incubated with different concentrations of NaCl, d-mannitol or urea (0, 74, or 134 mosmol/L added) for 6 (**left**) or 24 hours (**right**). In this experiment, concentrations are given as osmotic concentrations (mosmol/L) and not as molar concentrations (mmol/L). If no NaCl was added, the osmotic concentration corresponded to 103 mmol/L NaCl, 74 mosmol/L NaCl added to 140 mmol/L NaCl, and 134 mosmol/L NaCl added to 170 mmol/L NaCl. The mRNA expression of TonEBP and SMIT (sodium myo-inositol cotransporter) was measured by quantitative PCR (qPCR). NaCl and d-mannitol, which cause hypertonicity and cell shrinkage, upregulate TonEBP (**A**) and its downstream gene *SMIT* (**B**) more efficiently than urea, which causes hyperosmolarity but not hypertonicity and cell shrinkage. **C** and **D**, Different factors were knocked down or inhibited alone or combined in normal (103 mmol/L) and high NaCl (170 mmol/L) conditions in HTR-8/SVneo cells. Integrin β1 was knocked down by siRNA, focal adhesion kinase was inhibited by 10^−6^ M PF-573228, Src-family kinase by 10^−6^ M PP2, p38α/β by 10^−6^ M SB202190, EGFR (epidermal growth factor receptor) by 10^−6^ M AG1478 and G-protein coupled receptor 30 by 10^−6^ M G-15. The mRNA expression of TonEBP and SMIT was measured by qPCR. Inhibition of p38α/β diminished the SMIT increase at high NaCl more pronounced than ITGB1 (integrin β1) knockdown or inhibition of focal adhesion kinase, Src-family kinase, EGFR, or G-protein coupled receptor 30. Any combination of 2 inhibitors prevented the TonEBP and SMIT increase at high NaCl similar or even more than inhibition of p38α/β alone. Data are presented as mean±SD (n=3 biological replicates). In graphs **C** and **D** the symbol + written directly above the 170 mmol/L NaCl bars shows that 170 mmol/L compared with 103 mmol/L NaCl is significant. * or ^+^*P*<0.05, ** or ^++^*P*<0.01, *** or ^+++^*P*<0.001.

To differentiate whether hypertonicity or hyperosmolarity activates TonEBP in trophoblasts, diffusible and nondiffusable osmotic stimuli were tested. NaCl and d-mannitol are effective osmoles leading to extracellular hypertonicity and cell shrinkage, whereas urea is a highly diffusible osmolyte.^34^ HTR-8/SVneo cells were incubated with different concentrations of NaCl, d-mannitol, or urea. High NaCl (140–170 mmol/L NaCl) clearly upregulated *TonEBP* mRNA expression up to 3-fold at 6 hours, whereas the application of d-mannitol at low levels (74 mosmol/L) upregulated *TonEBP* mRNA expression up to 2-fold, even though this did not reach significance. High d-mannitol (134 mosmol/L added; *P*=0.071) and high urea (134 mosmol/L added; *P*=0.246) did not further upregulate *TonEBP* expression (Figure 4A). At 24 hours, no cellular responses were observed upon NaCl, d-mannitol, or urea stimulation in HTR-8/SV neo (Figure 4A). Hypertonicity, caused by high NaCl or d-mannitol, upregulated *SMIT* mRNA expression up to 10-fold after 6 hours. Conversely, hyperosmolarity mimicked by urea showed no effect on *SMIT* mRNA expression after 6 hours (Figure 4B). In contrast to *TonEBP* (Figure 4A), the elevated *SMIT* expression upon high NaCl, high d-mannitol, and high urea was significant at 24 hours (Figure 4B).

In summary, TonEBP was enhanced after osmotic challenge with NaCl, d-mannitol, and urea, but only translated into an SMIT signal in NaCl and d-mannitol, but not in the freely diffusible urea.

Consequently, factors activated by cytoskeleton signals were assessed, such as ITGB1 (integrin β1),^35^ focal adhesion kinase,^36^ Src-family kinases,^37^ p38α/β MAPK (mitogen-activated protein kinase),^38^ and EGFR (epidermal growth factor receptor).^39^ Inhibition or knockdown of these factors has been shown to prevent hypertonicity-induced TonEBP activation in other cell types. As these factors have not been tested for their relative impact in a pregnancy-specific cell type, HTR-8/SVneo cells were treated with ITGB1 siRNA, and inhibitors for focal adhesion kinase, Src-family kinase, p38α/β MAPK, and EGFR alone or in combination with normal (103 mmol/L) or high NaCl (170 mmol/L) conditions. As G-protein coupled receptors have been proposed to activate TonEBP,^40^ the inhibition of G-protein coupled receptors was additionally tested. Individual inhibition of these factors diminished the *TonEBP* increase at high NaCl exposure (Figure 4C). Either alone or in combination, the lowest *TonEBP* activation was observed in the presence of the p38α/β MAPK inhibitor (SB202190), suggesting this to be a key signaling step toward *TonEBP* expression upon osmotic challenge (Figure 4C). *SMIT* expression was not affected by inhibiting ITGB1 or the GPER1 (G protein-coupled estrogen receptor-1), whereas a strong and consistent reduction in *SMIT* expression was again observed upon inhibiting the p38α/β MAPK pathway (Figure 4D).

In summary, signaling related to cytoskeleton responses was involved in TonEBP and SMIT expression.

## DISCUSSION

In pregnancy, blood pressure is low despite plasma volume expansion and Na^+^ retention. In contrast, in preeclampsia, blood pressure is increased though intravascular volume is low.^41^ Likewise, Na^+^ retention is compromised in individuals destined to later develop preeclampsia.^42^

Increasing Na^+^ in pregnant women does not cause a rise in blood pressure.^12^ Nevertheless, uncertainty exists as to the potential active role of Na^+^ in promoting vasodilatory signals. Our novel data now show that during pregnancy a substantial placental Na^+^ retention occurs, which is reduced in preeclampsia. By simulating alterations in Na^+^ homeostatic conditions in trophoblasts, high Na^+^ exposure clearly augmented the tonicity signal TonEBP, resulting in enhanced proangiogenic signals (PlGF and VEGF-C). This response was driven by an altered transcellular osmotic gradient, as simulated by different osmolytes, via cytoskeletal-based signaling.

Consistent with earlier findings,^43^ maternal plasma renin and tetrahydroaldosterone concentrations are decreased in women with preeclampsia. As aldosterone synthesis in pregnancy is strongly driven by angiogenic signaling,^9,10^ antiangiogenesis in preeclampsia compromises aldosterone availability and thus Na^+^ retention.^44^

The placental Na^+^ content as assessed previously,^45^ was within a similar concentration range to those we observed in normal human placentae, yet the median content was lower in both early- and late-onset preeclampsia than in normotensive pregnancy. As either the renal Na^+^ retention, placental intracellular Na^+^ stores, and potentially to a much larger extent, the extracellular storage in compartments such as the cellular glycocalyx are conceivable, several mechanisms could be involved. A limitation was that we were not able to distinguish whether the Na^+^ concentrations were from intracellular pools or not, and future work is required to elucidate this. Moreover, our Na^+^/creatinine ratios were on spot urines and not 24-hour urines without control or assessment of patients’ dietary sodium intake and medication use, and treatments may have differed between patient groups before isolation of placentae. Moreover, when controlling for the use of antihypertensives, no differences in Na+ data were found. In line with the proposed Na^+^ availability in the placenta being critical, we demonstrated that both placenta and trophoblasts express markers of DCs characterizing tissues and cells responsive to Na^+^-induced signals.^5^ We confirm DC marker expression in our placental tissues (median [IQR], 9399 [2828–22 426] normalized copy number). TonEBP and SMIT expression has been described in the human placenta,^22^ being progressively upregulated throughout gestation, with the limitation that true physiological concentrations of NaCl were not used in these experiments. Accordingly, TonEBP was also dose dependently upregulated in trophoblasts upon Na^+^ exposure.

Of interest, placental expression of several Na^+^ channels was reported to be reduced in preeclampsia, such as epithelial Na^+^ channel and NHE-3 (sodium–hydrogen exchanger 3), a finding in line with the compromised aldosterone availability of preeclampsia.^46–48^ Strong redundancy of Na^+^-induced TonEBP activation was present in trophoblasts unaffected by inhibiting single Na^+^ channels, including epithelial Na^+^ channel. However, the activity of Na^+^/K^+^-ATPase was found to be critical for the maintenance of osmotic equilibrium, as inhibition of Na^+^/K^+^-ATPase in hypotonic conditions initially increased TonEBP but did not result in an SMIT signal.

While TonEBP regulation induced by changes in ambient tonicity has been known for some time in the kidney,^49^ other cell types, such as macrophages, have also been identified as contributing to the regulation of Na^+^ handling.^5^ In our study, in both established trophoblast cell lines and primary human term trophoblasts, hypertonic TonEBP regulation was paralleled by SMIT, via VEGF-C transcript expression. The regulation of the placental vascular endothelial growth factor homolog PlGF and Flt-1 showed upregulation on NaCl exposure in the cell lines but downregulation in the primary cells. In the latter, isolates with low VEGF-C expression demonstrated high PlGF levels and vice versa, suggesting a compensatory mechanism. However, we have previously reported that aldosterone, which is likely to enhance Na^+^ exposure via various mechanisms, upregulated PlGF.^50^

In contrast to observations of ouabain resistance of Na^+^ transport in the placenta,^51^ we have shown that TonEBP signaling was altered when Na^+^/K^+^-ATPase was inhibited. This is consistent with retention of intracellular Na^+^ as was proposed by Orlov and Hamet.^52^

The importance of a transmembrane osmotic gradient was underlined by an osmotic response to the nondiffusible osmolyte, d-mannitol, but not to diffusible urea. Consistent with a transmembrane osmotic difference affecting cytoskeletal changes by consecutive Ca^++^-activated p38α/β MAPK activation, its inhibition suppressed the osmotic signal, supporting the hypothesis that this pathway is central. Cytoskeletal signaling involved ITGB1 and transmembrane spanning receptors such as the epidermal growth factor receptor via Src-family and focal adhesion kinases. In line with other observations, our data suggest that TonEBP is regulated by receptor and integrin interaction such as the epithelial growth factor receptor.^53^ Of interest, our finding that ITGB1 enhances other osmotic stimuli in trophoblasts concurs with the original findings in the renal medulla.^35^

Tonicity changes affect the cytoskeleton in other cell types, and NaCl, mannitol, and urea have been implicated in those responses.^54^ We have now shown in trophoblasts that, while NaCl clearly enhanced TonEBP, the response was weaker with the other osmolytes. The nondiffusible mannitol elicited a vast early SMIT response, while the diffusible urea required long exposure to respond, contrasting with observations in renal MDCK cells.^54^

The first observation of abnormal placental Na^+^ handling in preeclampsia was made ≈70 years ago. Using ^24^Na normal saline as a tracer, a diminished rate of transfer of sodium across the placenta was observed in (toxemic) cases.^55^ We have now shown, for the first time, that the placental Na^+^ content is drastically lower in placentae from women with preeclampsia and that the Na^+^ environment is important for the regulation of trophoblast signaling relevant to maintain maternal endothelial integrity.

Overall, these observations open intriguing avenues, and we speculate that instead of restricting NaCl intake in pregnancies considered to be at risk of preeclampsia, a carefully monitored dietary increase of NaCl may possibly be considered as a potential early preventive strategy. However, further work is required before any such strategies are clinically introduced.

## PERSPECTIVES

In preeclampsia, women experience a depletion of placental Na^+^ reserves alongside decreased aldosterone levels and urinary sodium. This suggests a breakdown in the mechanism responsible for retaining salt and indicates potential abnormalities in Na^+^-related signals within the placenta. Additionally, when trophoblasts are exposed to higher levels of Na^+^, they exhibit increased expression of VEGF-C, a molecule known for its protective and vasodilatory effects on endothelial cells, mediated through TonEBP signaling. These findings highlight the placenta’s role as a previously unrecognized sensor of salt levels, complementing the role of the kidneys. It raises the possibility that maintaining an appropriate level of Na^+^ exposure in the placenta could be explored as a potential early preventive or therapeutic approach for preeclampsia.

## ARTICLE INFORMATION

### Acknowledgments

The authors thank all the research midwives/nurses, research practitioners, and clinical staff for help with recruitment. The authors also thank all the women who participated in the study.

### Sources of Funding

This work was produced by Hiten D. Mistry under the terms of a British Heart Foundation Basic Science Intermediate Basic Science Fellowship (FS/15/32/31604), the UK Research and Innovation Grand Challenges Research Fund GROW Award scheme (MR/P027938/1), and National Institute of Health Research–Wellcome Partnership for Global Health Research Collaborative Award (217123/Z/19/Z). Further support was by the Lindenhof Foundation, Berne, Switzerland.

### Disclosures

None.

### Supplemental Material

Expanded Materials & Methods

Tables S1–S4

Figures S1–S3

References 12, 26, 27, 43, and 50

## Supplementary Material



## References

[1] Steegers EA von Dadelszen P Duvekot JJ Pijnenborg R. Pre-eclampsia. Lancet. 2010;376:631–644. doi: 10.1016/S0140-6736(10)60279-6 20598363 10.1016/S0140-6736(10)60279-6

[2] Bellamy L Casas JP Hingorani AD Williams DJ. Pre-eclampsia and risk of cardiovascular disease and cancer in later life: systematic review and meta-analysis. BMJ. 2007;335:974. doi: 10.1136/bmj.39335.385301.BE 17975258 10.1136/bmj.39335.385301.BE PMC2072042

[3] Hytten F Leitch I. The Physiology of Human Pregnancy. 1st ed. Blackwell Scientific Publications; 1964.

[4] Salas SP Marshall G Gutierrez BL Rosso P. Time course of maternal plasma volume and hormonal changes in women with preeclampsia or fetal growth restriction. Hypertension. 2006;47:203–208. doi: 10.1161/01.HYP.0000200042.64517.19 16380519 10.1161/01.HYP.0000200042.64517.19

[5] Machnik A Neuhofer W Jantsch J Dahlmann A Tammela T Machura K Park JK Beck FX Muller DN Derer W. Macrophages regulate salt-dependent volume and blood pressure by a vascular endothelial growth factor-C-dependent buffering mechanism. Nat Med. 2009;15:545–552. doi: 10.1038/nm.1960 19412173 10.1038/nm.1960

[6] Gallery ED Brown MA. Volume homeostasis in normal and hypertensive human pregnancy. Baillieres Clin Obstet Gynaecol. 1987;1:835–851. doi: 10.1016/s0950-3552(87)80037-8 3330488 10.1016/s0950-3552(87)80037-8

[7] Duley L Henderson-Smart D Meher S. Altered dietary salt for preventing pre-eclampsia, and its complications. Cochrane Database Syst Rev. 2005;4:CD005548. doi: 10.1002/14651858.CD005548 10.1002/14651858.CD005548 PMC11270530 16235411

[8] Churchill D Beevers GD Meher S Rhodes C. Diuretics for preventing pre-eclampsia. Cochrane Database Syst Rev. 2007;2007:CD004451. doi: 10.1002/14651858.CD004451.pub2 17253507 10.1002/14651858.CD004451.pub2 PMC8826571

[9] Gennari-Moser C Khankin EV Escher G Burkhard F Frey BM Karumanchi SA Frey FJ Mohaupt MG. Vascular endothelial growth factor-A and aldosterone: relevance to normal pregnancy and preeclampsia. Hypertension. 2013;61:1111–1117. doi: 10.1161/HYPERTENSIONAHA.111.00575 23460276 10.1161/HYPERTENSIONAHA.111.00575

[10] Gennari-Moser C Khankin EV Schuller S Escher G Frey BM Portmann CB Baumann MU Lehmann AD Surbek D Karumanchi SA. Regulation of placental growth by aldosterone and cortisol. Endocrinology. 2011;152:263–271. doi: 10.1210/en.2010-0525 21068161 10.1210/en.2010-0525

[11] Mistry HD Kurlak LO Gardner DS Torffvit O Hansen A Broughton Pipkin F Strevens H. Evidence of augmented intrarenal angiotensinogen associated with glomerular swelling in gestational hypertension and preeclampsia: clinical implications. JAm Heart Assoc. 2019;8:e012611. doi: 10.1161/JAHA.119.012611 31237175 10.1161/JAHA.119.012611 PMC6662362

[12] Gennari-Moser C Escher G Kramer S Dick B Eisele N Baumann M Raio L Frey FJ Surbek D Mohaupt MG. Normotensive blood pressure in pregnancy: the role of salt and aldosterone. Hypertension. 2014;63:362–368. doi: 10.1161/HYPERTENSIONAHA.113.02320 24296282 10.1161/HYPERTENSIONAHA.113.02320

[13] Todkar A Di Chiara M Loffing-Cueni D Bettoni C Mohaupt M Loffing J Wagner CA. Aldosterone deficiency adversely affects pregnancy outcome in mice. Pflugers Archiv . 2012;464:331–343. doi: 10.1007/s00424-012-1145-4 22941338 10.1007/s00424-012-1145-4

[14] Farese S Shojaati K Kadereit B Frey FJ Mohaupt MG. Blood pressure reduction in pregnancy by sodium chloride. Nephrol Dial Transplant. 2006;21:1984–1987. doi: 10.1093/ndt/gfl106 16554323 10.1093/ndt/gfl106

[15] Robinson M. Salt in pregnancy. Lancet. 1958;1:178–181. doi: 10.1016/s0140-6736(58)90665-2 13503249 10.1016/s0140-6736(58)90665-2

[16] Eisele N Klossner R Escher G Rudloff S Larionov A Theilig F Mohaupt MG Mistry HD Gennari-Moser C. Physiological and molecular responses to altered sodium intake in rat pregnancy. J Am Heart Assoc. 2018;7:e008363. doi: 10.1161/JAHA.117.008363 30371243 10.1161/JAHA.117.008363 PMC6201473

[17] Pitkin RM Kaminetzky HA Newton M Pritchard JA. Maternal nutrition. A selective review of clinical topics. Obstet Gynecol. 1972;40:773–785. 4564722

[18] Oh YS Appel LJ Galis ZS Hafler DA He J Hernandez AL Joe B Karumanchi SA Maric-Bilkan C Mattson D. National Heart, Lung, and Blood Institute Working Group Report on salt in human health and sickness: building on the current scientific evidence. Hypertension. 2016;68:281–288. doi: 10.1161/HYPERTENSIONAHA.116.07415 27324228 10.1161/HYPERTENSIONAHA.116.07415 PMC4945367

[19] Rossitto G Bertoldi G Rutkowski JM Mitchell BM Delles C. Sodium, interstitium, lymphatics, and hypertension-A tale of hydraulics: salt series. Hypertension. 2024;81:727–737. doi: 10.1161/hypertensionaha.123.17942 38385255 10.1161/HYPERTENSIONAHA.123.17942 PMC10954399

[20] Wiig H Schroder A Neuhofer W Jantsch J Kopp C Karlsen TV Boschmann M Goss J Bry M Rakova N. Immune cells control skin lymphatic electrolyte homeostasis and blood pressure. J Clin Invest. 2013;123:2803–2815. doi: 10.1172/JCI60113 23722907 10.1172/JCI60113 PMC3696542

[21] Miyauchi H Geisberger S Luft FC Wilck N Stegbauer J Wiig H Dechend R Jantsch J Kleinewietfeld M Kempa S. Sodium as an important regulator of immunometabolism. Hypertension. 2024;81:426–435. doi: 10.1161/HYPERTENSIONAHA.123.19489 37675565 10.1161/HYPERTENSIONAHA.123.19489 PMC10863658

[22] Shin JA Kwon HM Han KH Lee HY. TonEBP and SMIT expression in human placenta. Anat Cell Biol. 2012;45:155–159. doi: 10.5115/acb.2012.45.3.155 23094203 10.5115/acb.2012.45.3.155 PMC3472141

[23] Ye BJ Lee HH Yoo EJ Lee CY Lee JH Kang HJ Jeong GW Park H Lee-Kwon W Choi SY. TonEBP in dendritic cells mediates pro-inflammatory maturation and Th1/Th17 responses. Cell Death Dis. 2020;11:421. doi: 10.1038/s41419-020-2632-8 32499518 10.1038/s41419-020-2632-8 PMC7272407

[24] Kammerer U Eggert AO Kapp M McLellan AD Geijtenbeek TB Dietl J van Kooyk Y Kampgen E. Unique appearance of proliferating antigen-presenting cells expressing DC-SIGN (CD209) in the decidua of early human pregnancy. Am J Pathol. 2003;162:887–896. doi: 10.1016/S0002-9440(10)63884-9 12598322 10.1016/S0002-9440(10)63884-9 PMC1868095

[25] Perez-Sepulveda A Torres MJ Khoury M Illanes SE. Innate immune system and preeclampsia. Front Immunol. 2014;5:244. doi: 10.3389/fimmu.2014.00244 24904591 10.3389/fimmu.2014.00244 PMC4033071

[26] Oh WC Mafrici B Rigby M Harvey D Sharman A Allen JC Mahajan R Gardner DS Devonald MAJ. Micronutrient and amino acid losses during renal replacement therapy for acute kidney injury. Kidney Int Rep. 2019;4:1094–1108. doi: 10.1016/j.ekir.2019.05.001 31440700 10.1016/j.ekir.2019.05.001 PMC6698297

[27] Nikitina L Wenger F Baumann M Surbek D Korner M Albrecht C. Expression and localization pattern of ABCA1 in diverse human placental primary cells and tissues. Placenta. 2011;32:420–430. doi: 10.1016/j.placenta.2011.03.003 21501868 10.1016/j.placenta.2011.03.003

[28] Kino T Takatori H Manoli I Wang Y Tiulpakov A Blackman MR Su YA Chrousos GP DeCherney AH Segars JH. Brx mediates the response of lymphocytes to osmotic stress through the activation of NFAT5. Sci Signal. 2009;2:ra5. doi: 10.1126/scisignal.2000081 19211510 10.1126/scisignal.2000081 PMC2856329

[29] Del Monaco SM Marino GI Assef YA Damiano AE Kotsias BA. Cell migration in BeWo cells and the role of epithelial sodium channels. J Membr Biol. 2009;232:1–13. doi: 10.1007/s00232-009-9206-0 19911219 10.1007/s00232-009-9206-0

[30] Orlov SN Koltsova SV Kapilevich LV Gusakova SV Dulin NO. NKCC1 and NKCC2: The pathogenetic role of cation-chloride cotransporters in hypertension. Genes Dis. 2015;2:186–196. doi: 10.1016/j.gendis.2015.02.007 26114157 10.1016/j.gendis.2015.02.007 PMC4477834

[31] Monroy A Plata C Hebert SC Gamba G. Characterization of the thiazide-sensitive Na(+)-Cl(-) cotransporter: a new model for ions and diuretics interaction. Am J Physiol Renal Physiol. 2000;279:F161–F169. doi: 10.1152/ajprenal.2000.279.1.F161 10894798 10.1152/ajprenal.2000.279.1.F161

[32] Kurita T Yamamura H Suzuki Y Giles WR Imaizumi Y. The ClC-7 chloride channel is downregulated by hypoosmotic stress in human chondrocytes. Mol Pharmacol. 2015;88:113–120. doi: 10.1124/mol.115.098160 25943117 10.1124/mol.115.098160

[33] Silva NL Wang H Harris CV Singh D Fliegel L. Characterization of the Na+/H+ exchanger in human choriocarcinoma (BeWo) cells. Pflugers Arch. 1997;433:792–802. doi: 10.1007/s004240050347 9049172 10.1007/s004240050347

[34] Santos BC Chevaile A Hebert MJ Zagajeski J Gullans SR. A combination of NaCl and urea enhances survival of IMCD cells to hyperosmolality. Am J Physiol. 1998;274:F1167–F1173. doi: 10.1152/ajprenal.1998.274.6.F1167 9841510 10.1152/ajprenal.1998.274.6.F1167

[35] Moeckel GW Zhang L Chen X Rossini M Zent R Pozzi A. Role of integrin alpha1beta1 in the regulation of renal medullary osmolyte concentration. Am J Physiol Renal Physiol. 2006;290:F223–F231. doi: 10.1152/ajprenal.00371.2004 16106035 10.1152/ajprenal.00371.2004

[36] Neuhofer W Kuper C Lichtnekert J Holzapfel K Rupanagudi KV Fraek ML Bartels H Beck FX. Focal adhesion kinase regulates the activity of the osmosensitive transcription factor TonEBP/NFAT5 under hypertonic conditions. Front Physiol. 2014;5:123. doi: 10.3389/fphys.2014.00123 24772088 10.3389/fphys.2014.00123 PMC3983490

[37] Cohen DM. SRC family kinases in cell volume regulation. Am J Physiol Cell Physiol. 2005;288:C483–C493. doi: 10.1152/ajpcell.00452.2004 15692147 10.1152/ajpcell.00452.2004

[38] Ko BC Lam AK Kapus A Fan L Chung SK Chung SS. Fyn and p38 signaling are both required for maximal hypertonic activation of the osmotic response element-binding protein/tonicity-responsive enhancer-binding protein (OREBP/TonEBP). J Biol Chem. 2002;277:46085–46092. doi: 10.1074/jbc.M208138200 12359721 10.1074/jbc.M208138200

[39] Kuper C Steinert D Fraek ML Beck FX Neuhofer W. EGF receptor signaling is involved in expression of osmoprotective TonEBP target gene aldose reductase under hypertonic conditions. Am J Physiol Renal Physiol. 2009;296:F1100–F1108. doi: 10.1152/ajprenal.90402.2008 19225051 10.1152/ajprenal.90402.2008

[40] Choi SY Lee-Kwon W Kwon HM. The evolving role of TonEBP as an immunometabolic stress protein. Nat Rev Nephrol. 2020;16:352–364. doi: 10.1038/s41581-020-0261-1 32157251 10.1038/s41581-020-0261-1

[41] Safar ME. Arterial stiffness as a risk factor for clinical hypertension. Nat Rev Cardiol. 2018;15:97–105. doi: 10.1038/nrcardio.2017.155 29022570 10.1038/nrcardio.2017.155

[42] Brown MA Nicholson E Gallery ED. Sodium-renin-aldosterone relations in normal and hypertensive pregnancy. Br J Obstet Gynaecol. 1988;95:1237–1246. doi: 10.1111/j.1471-0528.1988.tb06812.x 3066399 10.1111/j.1471-0528.1988.tb06812.x

[43] Kurlak LO Broughton Pipkin F Mohaupt MG Mistry HD. Responses of the renin-angiotensin-aldosterone system in pregnant chronic kidney disease patients with and without superimposed pre-eclampsia. Clin Kidney J. 2019;12:847–854. doi: 10.1093/ckj/sfz025 31807298 10.1093/ckj/sfz025 PMC6885683

[44] Asayama K Imai Y. The impact of salt intake during and after pregnancy. Hypertens Res. 2018;41:1–5. doi: 10.1038/hr.2017.90 29046520 10.1038/hr.2017.90

[45] Challier JC Bara M D’Athis P. The magnesium, calcium, sodium, potassium and chloride contents of the term human placenta. Magnes Res. 1988;1:141–145. 3275201

[46] Marino GI Kotsias BA. Expression of the epithelial sodium channel sensitive to amiloride (ENaC) in normal and preeclamptic human placenta. Placenta. 2013;34:197–200. doi: 10.1016/j.placenta.2012.11.008 23218889 10.1016/j.placenta.2012.11.008

[47] Dietrich V Szpilbarg N Damiano AE. Reduced expression of Na(+)/H(+) exchanger isoform 3 (NHE-3) in preeclamptic placentas. Placenta. 2013;34:828–830. doi: 10.1016/j.placenta.2013.06.005 23810111 10.1016/j.placenta.2013.06.005

[48] Speake PF Glazier JD Greenwood SL Sibley CP. Aldosterone and cortisol acutely stimulate Na+/H+ exchanger activity in the syncytiotrophoblast of the human placenta: effect of fetal sex. Placenta. 2010;31:289–294. doi: 10.1016/j.placenta.2009.12.025 20129665 10.1016/j.placenta.2009.12.025

[49] Neuhofer W Woo SK Na KY Grunbein R Park WK Nahm O Beck FX Kwon HM. Regulation of TonEBP transcriptional activator in MDCK cells following changes in ambient tonicity. Am J Physiol Cell Physiol. 2002;283:C1604–C1611. doi: 10.1152/ajpcell.00216.2002 12388086 10.1152/ajpcell.00216.2002

[50] Eisele N Albrecht C Mistry HD Dick B Baumann M Surbek D Currie G Delles C Mohaupt MG Escher G. Placental expression of the angiogenic placental growth factor is stimulated by both aldosterone and simulated starvation. Placenta. 2016;40:18–24. doi: 10.1016/j.placenta.2016.02.004 27016778 10.1016/j.placenta.2016.02.004

[51] Jacobs BE Liu Y Pulina MV Golovina VA Hamlyn JM. Normal pregnancy: mechanisms underlying the paradox of a ouabain-resistant state with elevated endogenous ouabain, suppressed arterial sodium calcium exchange, and low blood pressure. Am J Physiol Heart Circ Physiol. 2012;302:H1317–H1329. doi: 10.1152/ajpheart.00532.2011 22245773 10.1152/ajpheart.00532.2011 PMC3311474

[52] Orlov SN Hamet P. Salt and gene expression: evidence for [Na+]i/[K+]i-mediated signaling pathways. Pflugers Arch. 2015;467:489–498. doi: 10.1007/s00424-014-1650-8 25479826 10.1007/s00424-014-1650-8

[53] Halterman JA Kwon HM Wamhoff BR. Tonicity-independent regulation of the osmosensitive transcription factor TonEBP (NFAT5). Am J Physiol Cell Physiol. 2012;302:C1–C8. doi: 10.1152/ajpcell.00327.2011 21998140 10.1152/ajpcell.00327.2011 PMC3328893

[54] Kwon MS Na KY Moeckel G Lee SD Kwon HM. Urea promotes TonEBP expression and cellular adaptation in extreme hypertonicity. Pflugers Archiv. 2009;459:183–189. doi: 10.1007/s00424-009-0696-5 19585141 10.1007/s00424-009-0696-5

[55] Cox LW Chalmers TA. The effect of pre-eclamptic toxaemia on the exchange of sodium in the body and the transfer of sodium across the placenta, measured by Na24 tracer methods. J Obstet Gynaecol Br Emp. 1953;60:214–221. doi: 10.1111/j.1471-0528.1953.tb07677.x 13053285 10.1111/j.1471-0528.1953.tb07677.x

