# Supplementary material for: Alterations of Placental Sodium in Preeclampsia: Trophoblast Responses
Source: Hypertension. 2024 Jul 5;81(9):1924–34. doi: 10.1161/HYPERTENSIONAHA.124.23001 (PMC11319085; doi:10.1161/HYPERTENSIONAHA.124.23001)
Supplement: Supplementary file 1 [file hyp-81-1924-s001.doc]

**Supplementary data**

**Alterations of placental sodium in pre-eclampsia - trophoblast responses**

Hiten D. Mistry PhD ^1,#^, Rahel Klossner BSc ^2,3,4 #^ , Paula J. Scaife PhD ^5^, Nicole Eisele PhD ^3,4^, Lesia O. Kurlak PhD^6^, Sampada Kallol PhD ^7^, Christiane Albrecht PhD ^7^, Carine Gennari-Moser PhD ^3,4^, Louise V. Briggs BSc ^8^, Fiona Broughton Pipkin DPhil ^9^, Markus G. Mohaupt PhD ^2,3,4*^

*^1^Department of Women and Children’s Health, School of Life Course and Population Health Sciences, King’s College London, London, UK; ^2^Teaching Hospital Internal Medicine, Lindenhofgruppe, 3006 Bern, Switzerland; ^3^Department of Nephrology and Hypertension, University of Bern, 3010 Bern, Switzerland; ^4^Department for BioMedical Research, University of Bern, 3010 Bern, Switzerland; ^5^Clinical, Metabolic and Molecular Physiology, University of Nottingham, Nottingham, UK; ^6^ Stroke Trials Unit (School of Medicine), University of Nottingham, Nottingham, UK; ^7^Institute for Biochemistry and Molecular Medicine, University of Bern, 3012 Berne, Switzerland; ^8^Advanced Material Research Group, Faculty of Engineering, University of Nottingham, Nottingham, UK; ^9^Department of Obstetrics, City Hospital Nottingham, Nottingham, Nottingham, UK*

^#^authors made equal contribution

**Short Title:** The placenta as a novel salt sensor

***Corresponding Author:**

Hiten D. Mistry BSc (Hons); PhD

Department of Women & Children’s Health

School of Life Course and Population Health Sciences,

King’s College London, London, UK.

Tel. +44(0)20 7848 9571

Email: [hiten.mistry@kcl.ac.uk](mailto:hiten.mistry@kcl.ac.uk)

**Materials and Methods**

*Patients*

Fully informed, signed consent for participation was obtained from all women, following HRA-REC ethics committee approval of the study gained by the University of Nottingham (REF: 15/EM/0523). All procedures involving the participants were in accordance with the Helsinki Declaration of 1975. All women were of white, Western European ethnicity. Pre-eclampsia (n=50) was defined as systolic blood pressure >140 mm Hg and diastolic blood pressure >90 mmHg, determined on 2 occasions >4 hours apart and arising after 20 weeks of gestation in a previously normotensive woman. This was accompanied by *de novo* proteinuria (protein: creatinine ratio (PCR) > 30; urine protein concentration > 3 g/L in 2 random clean-catch midstream specimens collected >4 hours apart) with no evidence of urinary tract infection. No women had any underlying renal or hypertensive disease before 20 weeks of gestation. For sub-group analysis, the pre-eclampsia group was further split by early- (diagnosis ≤ 34 weeks; n=21) and late- (diagnosis > 34 weeks; n=29)) onset pre-eclampsia.

Healthy normotensive pregnant women (n=55) matched for age were also recruited, who had no complications and no evidence of any urinary tract infections. Finally, healthy non-pregnant women (n=26), age-matched with the pregnant women were recruited. Medical and obstetric histories, including delivery data, were obtained from each woman. A summary of the demographic and pregnancy outcome of the women recruited in this study is presented in Table 1.

Maternal venous blood samples and urine were collected before delivery; where possible, umbilical venous (fetal) blood samples were collected immediately after delivery. Samples were processed as previously described^1^ and stored as 250 μl aliquots at -80^o^C until analysis. In addition, full-depth placental tissue biopsies were collected within 10 mins of the placenta being delivered. Samples were collected from a standardised location midway between the cord insertion and placental border and either snap frozen for gene expression or processed for immunohistochemistry as previously described^1^.

*Measurement of Na^+^ content in human plasma, urine and placenta*

Plasma and urine concentrations of Na^+^ were assayed by Inductively Coupled Plasma Mass Spectrometry (ICP-MS). Samples and standards (SPEX Certiprep Inc.) were prepared identically in a diluent containing 0.1% ‘Triton X-100’ non-ionic surfactant (+‘antifoam-B’, Sigma), 2% methanol and 1% HNO_3_ (trace analysis grade) including the internal ICP-MS standards Iridium (5 µg/L), Rhodium (10 µg/L), Gallium (25 µg/L) and Scandium (50 µg/L). The ICP-MS was run in collision-reaction cell mode for all analytes, with pure H_2_ as the cell gas to maximise sensitivity. Aspiration was through a single sample line via a Burgener-Miramist PEEK nebuliser. Calibrations for Na^+^ were in the range 0 – 50 µg/L. Quality of analysis was assured by the use of the reference materials Seronorm and UTAK (Nycomed Pharma AS); limits of detection have been published previously^2^.

Placental tissue concentrations of Na^+^ and K^+^ (mmol/g of dry matter (DM)), were also measured by ICP-MS (intra-assay variability <2%), after prior digestion of ~400 mg of freeze-dried tissue to determine the percentage of water content and subsequent digestion using 2% nitric acid. Certified reference material (NIST SRM bovine liver, 1577c) was used to validate elemental recovery and to correct for any batch variation.

*RAAS assays*

Plasma active renin concentrations were assayed as previously described^1^. In brief, samples were measured blinded to group/outcome, in duplicate, by enzyme-linked immunosorbent assay (ELISA) (IBL, Hamburg, Germany). Intra- and inter-assay coefficients of variation were 4.1 % and 8.1%, respectively.

Plasma and urine AGT concentrations were measured in duplicate and blinded to group/outcome as described earlier^1^. Plasma samples were diluted 1:10,000 and urine samples between 1:4 and 1:30, using buffer provided within the ELISA kit (IBL, Japan). Intra- and inter-assay coefficients of variation were 3.6 % and 6.2 %, respectively.

Urine tetrahydroaldosterone (TH-aldo), the major metabolite of aldosterone was assayed by gas chromatography-mass spectrometry (GC-MS), according to a method applied by our group as reported earlier^1^. TH-aldo was measured rather than native aldosterone since the mass spectrometry-based method accurately measures individual steroid hormone concentrations. Many conventional assays for aldosterone, such as ELISAs and radioimmunoassays, are compromised by significant cross-reactivity with progesterone and lack of specificity^1^. Details of sample preparation can be found in our previous publications^1^.

*Urinary creatinine concentrations*

Creatinine was measured in undiluted urine samples (500 µl) using an automated assay, based on the enzymatic reaction of creatinine with picric acid producing a colormetric product (RX-Imola; Randox Laboratories Ltd, London, UK).

All urine measurements were then normalised to creatinine. An interpretation of how urine Na^+^/creatinine measurements is associated with approximate dietary sodium intakes per day per day was made. This was calculated from our prior well controlled study^3^ where pregnant women excreted on average 19 mmol Na^+^/mmol creatinine in the urine, which roughly corresponds to 13 g NaCl/d in the first trimester. In these third trimester measurements presented here, the intake in the non-pre-eclamptic group corresponds to 8-9 g/d, in the pre-eclamptic patients 3-4 g/d. However, the latter could be compromised by volume depletion due to interstitial losses and oedema formation related to the endothelial dysfunction present at this time.

*RNA extraction and quantitative real-time PCR (qPCR)*

Total RNA was extracted from rat and human placenta and cells by means of TriZOL reagent (Thermo Fisher Scientific). RNA concentration was quantified using NanoDrop ND-1000. RNA (0.3-1 µg), were reverse transcribed with PrimeScript™ RT Reagent Kit (#RR037A Takara Bio Inc.). For qPCR, primer pairs (Table S1) were designed using the Universal ProbeLibrary Assay Design Center software from Roche. All primers were intron-spanning and did not amplify genomic DNA. Primers were synthesized by Microsynth AG (Balgach, Switzerland) and Universal ProbeLibrary hydrolysis probes were purchased from Sigma-Aldrich. qPCR reactions (12.5ng cDNA/reaction, 200nM primer, 100nM probe) were performed in duplicate using Taqman Fast Universal PCR Master Mix (#4352046 Thermo Fisher Scientific) and measured with 7500 FAST Real-Time PCR System (Thermo Fisher Scientific). The amplification profile used was: 95°C 20sec, 95°C 3sec, 60°C 30sec. A no-reverse transcriptase control (NRT) and a no-template control (NTC) were included. The NRTs had a quantification cycle (Cq) which was at least 10 cycles higher than the Cq of the samples; the NTCs had an undetected Cq. Mean quantification cycles are shown in Table S2. Cell line data were normalized to the reference gene cyclophilin A (#4326316E Thermo Fisher Scientific), primary cell data to the geometric mean of cyclophilin A, HPRT1 and YWHAZ and rat placenta data to the geometric mean of YWHAZ, HPRT1 and HMBS. All results are presented as fold-change (2^-ΔΔCT^) relative to the mean of the control.

*Western blot*

Proteins were extracted with sucrose buffer (250 mM Sucrose, 10 mM Tris Base pH 7.5, cOmplete™ Mini Protease Inhibitor Cocktail (#11836153001 Sigma-Aldrich), 1 mM phenylmethanesulfonyl fluoride, 10 mM sodium orthovanadate). After 15 min centrifugation at 13’000 rpm and 4°C, the supernatant was collected. The total protein amount was measured with the Pierce BCA Protein Assay Kit (#23227 Thermo Fisher Scientific). Equal amounts of total protein were separated on an 8% sodium dodecyl sulfate polyacrylamide gel under reducing conditions and electroblotted onto a 0.45 µm PVDF membrane (#10600023 Sigma-Aldrich). Total protein was visualized by Ponceau S staining (#P7170 Sigma-Aldrich). Then the blots were blocked with 5% milk in TBST (TBS containing 0.1% Tween 20) for 1hr at room temperature and incubated overnight at 4°C with anti-TonEBP (#ab3446 Abcam, 1 µg/ml in TBST) or anti-β-actin (#sc-69879 Santa Cruz, 0.1 µg/ml in 5% milk/TBST). After four washes with TBST, the blots were incubated for 1hr at room temperature with horseradish peroxidase-conjugated secondary antibody (#sc-2004 or #sc-2314 Santa Cruz, 0.04 µg/ml in TBST or 5% milk/TBST). An enhanced chemiluminescence system (#RPN2106 Sigma-Aldrich) was used to visualize the antibody binding and images were acquired using the ChemiDoc^TM^ XRS+ system. Band intensity was quantified using ImageJ and normalized to β-actin or Ponceau S staining (bands between 35 and 55 kDa). To compare the biological replicates on different blots, one control sample was loaded onto every blot and used for normalisation. TonEBP band specificity was confirmed with the immunizing peptide (#ab4978 Abcam).

*Cell culture material*

The human choriocarcinoma cell lines BeWo (CCL98™) and JEG-3 (HTB36™) were obtained from American Type Culture Collection (Manassas, USA). The human first-trimester trophoblast cell line HTR-8/SVneo was a gift from Charles H. Graham (Queen’s University, Kingston, Ontario, Canada). Cell culture plates were from Corning Inc. (Amsterdam, Netherlands): for JEG-3 and HTR-8/SVneo tissue culture treated plates were used; for cytotrophoblasts (CTBs) and BeWo collagen I-coated plates were used. All cell culture media and supplements were obtained from Thermo Fisher Scientific Inc. (Reinach, Switzerland). BeWo cells were cultured in DMEM/F12K medium (#41965 and #21127 1:1), JEG-3 in McCoy’s medium (#36600), HTR-8/SVneo in RPMI 1640 medium (#21875) and CTBs in DMEM (#41965). Media were supplemented with 10% FBS, respectively 5% FBS for HTR-8/SVneo, 100 units/mL penicillin and 100 µg/mL streptomycin. Additionally, medium for CTBs contained 0.25 µg/mL Amphotericin B. Cells were cultured at 37°C and 5% CO_2_. BeWo cells were used from passage 22 to 35, JEG-3 from passage 15 to 28, and HTR-8/SVneo from passage 89-101. Primary term trophoblasts were used directly after isolation. All cells were used at subconfluence.

The inhibitor G-15 (#3678, GPER1 antagonist) was purchased from Bio-Techne AG (Zug, Switzerland). The inhibitors PF-573228 (#PZ0117, FAK inhibitor), PP2 (#P0042, Src family kinase inhibitor), SB202190 (#S7067, p38α and -β inhibitor) and Tyrphostin AG1478 (#T4182, EGFR inhibitor), ouabain (#O3125, Na/K-ATPase inhibitor), amiloride (#A7410, ENaC inhibitor), furosemide (#F4381, NKCC inhibitor), chlorothiazide (#C4911, NCC inhibitor), disodium 4,4′-diisothiocyanatostilbene-2,2′-disulfonate (DIDS, #D3514, anion exchanger inhibitor), 5-(N,N-hexamethylene)amiloride (HMA, #A9561, NHE inhibitor) were obtained from Sigma-Aldrich (Buchs, Switzerland). Sodium chloride (NaCl), D-mannitol, urea, MTT and DMSO were also obtained from Sigma-Aldrich. Silencer® Select validated ITGB1 siRNA (#s7574), Silencer® Select validated Brx siRNA (#s680), Silencer® Select negative control siRNA #1 (#4390843), Lipofectamine RNAiMAX (#13778) and Opti-MEM (#31985) were purchased from Thermo Fisher Scientific. The media used contained between 103 and 120 mM NaCl (Table S3). This corresponds to 139-158 mM Na^+^, because the media not only contained Na^+^ in the form of NaCl, but also in the form of sodium bicarbonate and sodium phosphate. Medium osmolarity was increased by addition of NaCl, D-mannitol or urea. 20-67 mM NaCl were added to reach a final NaCl concentration of 140 or 170 mM. As NaCl dissociates in solution into Na^+^ and Cl^-^, 1 mM NaCl corresponds to 2 mosmol/l. As D-mannitol and urea do not dissociate, 1 mM corresponds to 1 mosmol/l. To reach the same osmotic concentration, the added molar concentration of D-mannitol or urea was twice the molar concentration of NaCl.

*Isolation of human primary term trophoblasts*

Human primary term trophoblasts were isolated from term placentae (38-40 weeks of gestation) from healthy, normotensive pregnant women following informed consent. The isolation procedure was as previously described^4^. Primary cytotrophoblasts were cultured as previously reported^5^ in DMEM:F12K (1:1), containing 10% FBS penicillin, streptomycin and amphotericin B and plated on collagen I-coated culture dishes. Cells were cultured at 37^O^C and 5% CO_2_.

*Cell culture experiment with different NaCl, D-mannitol or urea concentrations*

BeWo, JEG-3 and HTR-8/SVneo cells were cultured for 24 hrs in their corresponding medium containing FBS. After 24hrs, cells were starved in medium with reduced serum 0.1% FBS. After 24 hrs, medium was replaced (0.1% FBS) and the indicated concentrations of NaCl, D-mannitol or urea were added for 1-24 hrs.

Primary term trophoblasts were cultured for 16 hrs. Thereafter, medium was replaced (10% FBS) and the indicated concentrations of NaCl were added for 6 or 24 hrs. No starving step was performed as primary cells are very sensitive to low serum conditions.

At the end of the experiment, cells were washed with phosphate-buffered saline (PBS) and RNA or protein extraction was done as described above.

*Cell culture experiment with ITGB1 knockdown and inhibitors of FAK, Src family kinase, p38*α/β*, EGFR, GPR30*

ITGB1 siRNA or negative control siRNA were reverse transfected into HTR-8/SVneo by means of Lipofectamine RNAiMAX. Negative control siRNA, which does not target any gene product, was used to control for the effect of siRNA delivery. To achieve this, siRNA and Lipofectamine were diluted in Opti-MEM directly in the wells of a cell culture plate and incubated for 20min. Then, HTR-8/SVneo cells were added to the wells and incubated for 24hrs. The final concentrations were 2 nM siRNA, 2 µl/ml Lipofectamine and 200 µl/ml Opti-MEM. After 24hrs of transfection, cells were washed with medium and incubated for 1hr in medium (0.1% FBS) with inhibitors. Cells were then incubated for an additional 6 hrs in medium (0.1% FBS) with inhibitors and the indicated NaCl concentrations. Thereafter, cells were washed with PBS and RNA was extracted as described. The stock solutions of the inhibitors were prepared in dimethyl sulphoxide (DMSO), the final DMSO concentration in medium was 0.1% and the final concentration of all inhibitors in medium was 10^-6^ M. Medium without penicillin and streptomycin was used throughout.

*Cell culture experiment with different NaCl-transporter inhibitors*

BeWo cells were cultured for 24hrs and then starved for 24 hrs in medium containing 0.1% FBS. Thereafter, the NaCl-transporter inhibitors amiloride, furosemide, chlorothiazide, DIDS and HMA were added for 1hr. After the 1hr pre-incubation period. Cells were stimulated with the indicated NaCl concentrations and the inhibitors in serum-depleted medium (0.1% FBS) for additional 6hrs. After 6hrs, cells were washed with PBS and RNA was extracted as described. The stock solutions of the NaCl-transporter inhibitors were prepared in DMSO and the final DMSO concentration in medium was 0.1%. The final concentration of the inhibitors on the cells was 10^-4^ M oubain, 10^-5^ M amiloride, 10^-4^ M furosemide, 10^-4^ M chlorothiazide, 10^-4^ M DIDS and 10^-6^ M HMA.

*Cell culture experiment with Brx knockdown*

Brx siRNA or negative control siRNA were reverse transfected into HTR-8/SVneo using Lipofectamine RNAiMAX, as described above for ITGB1 siRNA. 24hrs after transfection, cells were stimulated with the indicated NaCl concentrations in serum-depleted medium (0.1% FBS) for 6hrs. At the end of the incubation period, cells were washed with PBS and RNA was extracted as described. Medium without penicillin and streptomycin was used throughout.

*MTT assay*

Cell viability was assessed by the MTT assay. Cells were cultured in triplicates in 96-well plates and treated according to the experiments described above. At the end of the experiment, the medium was removed, 100 μl MTT solution (0.5 mg/ml MTT in medium) was added per well for 30-60 min at 37°C. After 30-60min, the MTT solution was removed and 150 μl DMSO and 19 μl Sorenson solution (0.1 M glycine, 0.1 M NaCl, pH 10.5) were added to each well. Absorbance was measured at 570 nm and the blank absorbance (no cells) was subtracted. Viability was calculated as percent of the viability of the control condition.

**References**

1. Kurlak LO,Broughton Pipkin F,Mohaupt MG, Mistry HD. Responses of the renin-angiotensin-aldosterone system in pregnant chronic kidney disease patients with and without superimposed pre-eclampsia. *Clin Kidney J*. 2019;12:847-854. doi: 10.1093/ckj/sfz025

2. Oh WC,Mafrici B,Rigby M,Harvey D,Sharman A,Allen JC,Mahajan R,Gardner DS, Devonald MAJ. Micronutrient and Amino Acid Losses During Renal Replacement Therapy for Acute Kidney Injury. *Kidney Int Rep*. 2019;4:1094-1108. doi: 10.1016/j.ekir.2019.05.001

3. Gennari-Moser C,Escher G,Kramer S,Dick B,Eisele N,Baumann M,Raio L,Frey FJ,Surbek D, Mohaupt MG. Normotensive blood pressure in pregnancy: the role of salt and aldosterone. *Hypertension*. 2014;63:362-368. doi: 10.1161/HYPERTENSIONAHA.113.02320

4. Nikitina L,Wenger F,Baumann M,Surbek D,Korner M, Albrecht C. Expression and localization pattern of ABCA1 in diverse human placental primary cells and tissues. *Placenta*. 2011;32:420-430. doi: 10.1016/j.placenta.2011.03.003

5. Eisele N,Albrecht C,Mistry HD,Dick B,Baumann M,Surbek D,Currie G,Delles C,Mohaupt MG,Escher G, et al. Placental expression of the angiogenic placental growth factor is stimulated by both aldosterone and simulated starvation. *Placenta*. 2016;40:18-24. doi: 10.1016/j.placenta.2016.02.004

**Table S1**

Human primers used in qPCR

| **Gene** | **Accession number** | **Primers** | **Probe** | **Amplicon size (base pairs)** |
| --- | --- | --- | --- | --- |
|  |  |  |  |  |
| TonEBP | NM_138714.3 | 5'- tct cat gat gtt caa cca ttc ac -3’  5'- cac att tac att caa agc acc ag -3’ | 3 | 69 |
|  |  |  |  |  |
| SMIT | NM_006933.6 | 5'- cgc tac gag ctg gct tta at -3’  5'- tcc aca aga cca tca gca tt -3’ | 25 | 124 |
|  |  |  |  |  |
| VEGF-C | NM_005429.4 | 5'- atg cct ggc tca gga aga t -3’  5'- ttg gtc cac aga tgt cat gg -3’ | 67 | 83 |
|  |  |  |  |  |
| PlGF | NM_002632.5 | 5'- gtt cag ccc atc ctg tgt ct -3’  5'- ccc aga acg gat ctt tag ga -3’ | 66 | 121 |
|  |  |  |  |  |
| Flt-1 | NM_002019.4 | 5'- cca ctc cct tga aca cga g -3’  5'- gtc gcc tta cgg aag ctc t -3’ | 85 | 78 |
|  |  |  |  |  |
| Brx | NM_006738.5 | 5'- tgt cca gag cgt tgt tca tc -3’  5'- tgg tcc tca atg tag ctg tcc -3’ | 73 | 84 |
|  |  |  |  |  |
| ITGB1 | NM_002211.3 | 5'- cga tgc cat cat gca agt -3’  5'- aca cca gca gcc gtg taa c -3’ | 65 | 71 |
|  |  |  |  |  |
| HPRT1 | NM_000194.2 | 5'- tga cct tga ttt att ttg cat acc -3’  5'- cga gca aga cgt tca gtc ct -3’ | 73 | 102 |
|  |  |  |  |  |
| YWHAZ | NM_003406.3 | 5'- gca att act gag aga caa ctt gac a -3’  5'- tgg aag gcc ggt taa ttt t -3’ | 2 | 96 |

**Table S2**

Mean quantification cycles of the genes measured by qPCR

| gene | **HTR-8/SVneo** | **JEG-3** | **BeWo** | **CTB** | **Rat placenta** |
| --- | --- | --- | --- | --- | --- |
| **TonEBP** | 28 | 28 | 27 | 26 | 26 |
| **SMIT** | 29 | 29 | 28 | 29 | 28 |
| **VEGF-C** | 24 | >35 | >35 | 33 | 31 |
| **PlGF** | 31 | 26 | 25 | 26 | 27 |
| **Flt-1** | 25 | 33 | >35 | 19 | 25 |

Mean of the control conditions is shown for the cells, and mean of NS diet for rat placenta

**Table S3**

NaCl, Na^+^ and Cl^-^ concentrations in cell culture media

| **mM NaCl** | **mM NaCl added** | **mosmol/l added** | **mM Na^+^** | **mM Cl^-^** |
| --- | --- | --- | --- | --- |
| HTR-8/SVneo medium |  |  |  |  |
| 103 | 0 | 0 | 139 | 109 |
| 140 | 37 | 74 | 176 | 146 |
| 170 | 67 | 134 | 206 | 176 |
| JEG-3 medium |  |  |  |  |
| 111 | 0 | 0 | 142 | 118 |
| 140 | 29 | 58 | 171 | 147 |
| 170 | 59 | 118 | 201 | 177 |
| BeWo medium |  |  |  |  |
| 120 | 0 | 0 | 158 | 128 |
| 140 | 20 | 40 | 178 | 148 |
| 170 | 50 | 100 | 208 | 178 |
| CTB medium |  |  |  |  |
| 110 | 0 | 0 | 155 | 119 |
| 140 | 30 | 60 | 185 | 149 |
| 170 | 60 | 120 | 215 | 179 |

**Table S4**

Cell viability after different cell culture treatments measured by MTT assay

| HTR-8/SVneo |  |  |  |
| --- | --- | --- | --- |
| time | **103mM NaCl** | **140mM NaCl** | **170mM NaCl** |
| **1h** | 100±0 | 98±3 | 96±4 |
| **2h** | 100±0 | 94±3 | 88±6 |
| **3h** | 100±0 | 96±1 | 92±0 |
| **6h** | 100±0 | 98±1 | 92±4 |
| **8h** | 100±0 | 99±3 | 94±4 |
| **16h** | 100±0 | 98±4 | 83±1 |
| **24h** | 100±0 | 106±12 | 102±13 |
| JEG-3 |  |  |  |
| time | **111mM NaCl** | **140mM NaCl** | **170mM NaCl** |
| **1h** | 100±0 | 95±10 | 100±11 |
| **2h** | 100±0 | 97±2 | 97±5 |
| **3h** | 100±0 | 97±4 | 97±5 |
| **6h** | 100±0 | 95±2 | 104±6 |
| **8h** | 100±0 | 93±3 | 102±5 |
| **16h** | 100±0 | 92±1 | 81±6 |
| **24h** | 100±0 | 110±8 | 99±10 |
| BeWo |  |  |  |
| time | **120mM NaCl** | **140mM NaCl** | **170mM NaCl** |
| **1h** | 100±0 | 105±9 | 109±21 |
| **2h** | 100±0 | 96±3 | 88±7 |
| **3h** | 100±0 | 99±1 | 93±6 |
| **6h** | 100±0 | 101±6 | 86±5 |
| **8h** | 100±0 | 93±4 | 76±7 |
| **16h** | 100±0 | 112±9 | 83±13 |
| **24h** | 100±0 | 106±17 | 65±19 |
| CTB |  |  |  |
| time | **110mM NaCl** | **140mM NaCl** | **170mM NaCl** |
| **6h** | 100±0 | 93±5 | 79±7 |
| **24h** | 100±0 | 94±6 | 73±10 |
| HTR-8/SVneo |  |  |  |
| osmolyte | **0mosmol/l added** | **74mosmol/l added** | **134mosmol/l added** |
| **NaCl** | 100±0 | 108±3 | 101±1 |
| **D-mannitol** | 100±0 | 98±1 | 99±4 |
| **Urea** | 100±0 | 103±4 | 93±4 |
| HTR-8/SVneo |  |  |  |
| siRNA or inhibitor | **103mM NaCl** | **170mM NaCl** |  |
| **control** | 100±0 | 101±3 |  |
| **ITGB1 siRNA** | 96±2 | 97±3 |  |
| **PF-573228** | 105±3 | 100±11 |  |
| **PP2** | 96±4 | 89±11 |  |
| **SB202190** | 101±3 | 90±12 |  |
| **AG1478** | 106±2 | 100±5 |  |
| **ITGB1 siRNA + PF-573228** | 97±3 | 93±5 |  |
| **ITGB1 siRNA + PP2** | 95±7 | 88±6 |  |
| **ITGB1 siRNA + SB202190** | 94±5 | 84±6 |  |
| **ITGB1 siRNA + AG1478** | 93±6 | 94±7 |  |
| **PF-573228 + PP2** | 98±0 | 100±5 |  |
| **PF-573228 + SB202190** | 104±2 | 92±11 |  |
| **PF-573228 + AG1478** | 102±5 | 100±7 |  |
| **PP2 + SB202190** | 100±5 | 93±7 |  |
| **PP2 + AG1478** | 97±3 | 96±4 |  |
| **SB202190 + AG1478** | 101±3 | 100±4 |  |
| **G-15** | 95±5 | 97±4 |  |
| HTR-8/SVneo |  |  |  |
| siRNA | **103mM NaCl** | **140mM NaCl** | **170mM NaCl** |
| **Negative control siRNA** | 100±0 | 107±5 | 107±4 |
| **Brx siRNA** | 95±1 | 97±5 | 106±4 |
| BeWo |  |  |  |
| inhibitor | **120mM NaCl** | **170mM NaCl** |  |
| **DMSO control** | 100±0 | 75±5 |  |
| **Ouabain** | 37±25 | 41±15 |  |
| **Amiloride** | 97±3 | 83±5 |  |
| **Furosemide** | 104±5 | 86±4 |  |
| **Chlorothiazide** | 107±5 | 82±3 |  |
| **DIDS** | 86±4 | 70±5 |  |
| **HMA** | 91±8 | 75±4 |  |

Mean±SD is shown; n=6 for CTB; n=3 for HTR-8/SVneo, JEG-3 and BeWo.

**
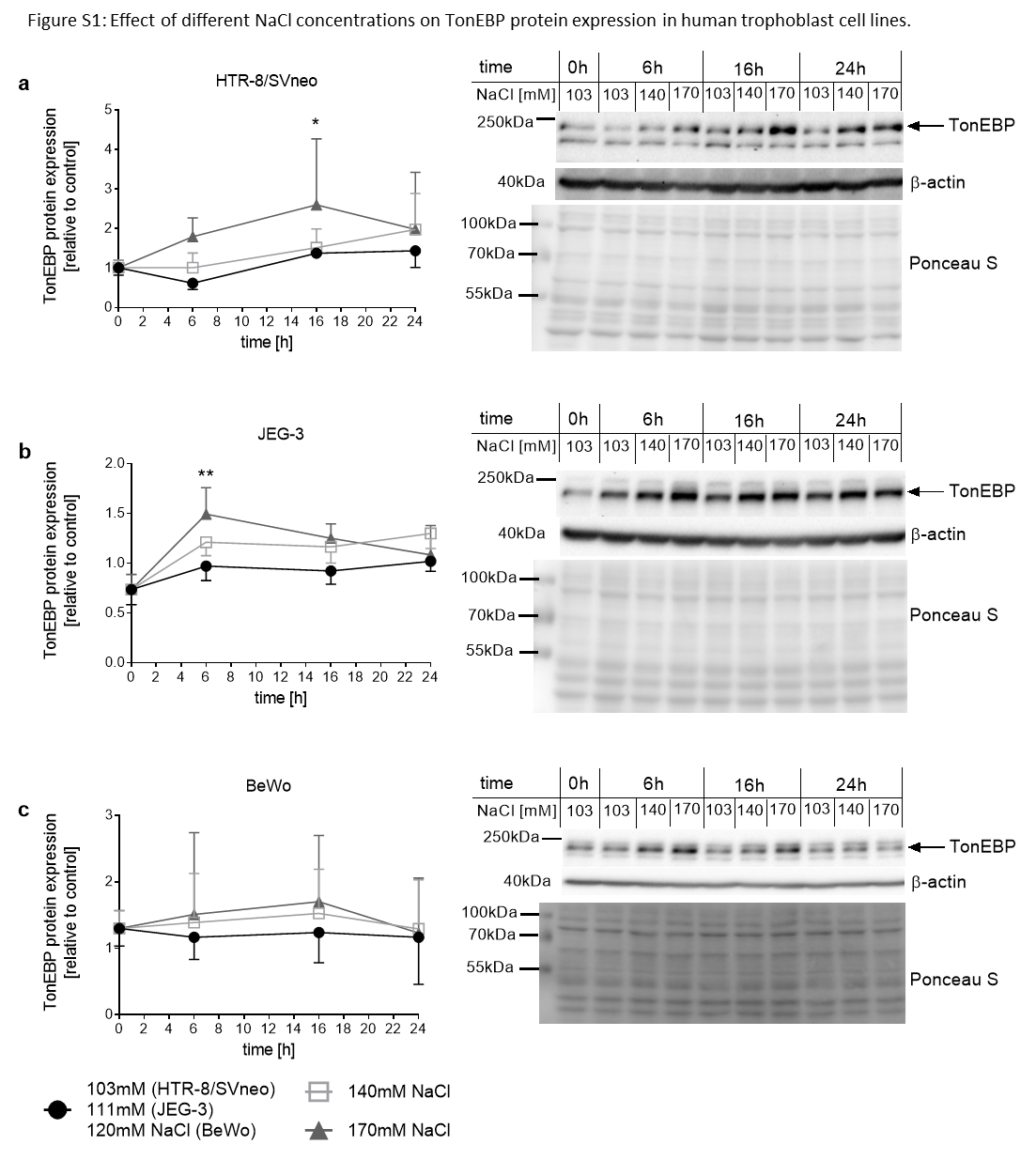
**

**Supplementary Figure S1:** Effect of different NaCl concentrations on TonEBP protein expression in human trophoblast cell lines. A) HTR-8/SVneo, B) JEG-3 and C) BeWo were incubated with normal (103 mM for HTR-8/SVneo, 111 mM for JEG-3, 120 mM for BeWo) or high NaCl concentrations (140 mM, 170 mM) for 0, 6, 16 or 24 hrs. The protein expression of TonEBP was measured by western blot. TonEBP band intensity was normalised to Ponceau S staining (bands between 35 and 55kDa) or β-actin. Both normalisations led to similar results and therefore only normalisation to Ponceau S is shown (left). High NaCl increased TonEBP protein expression in all cell lines, although not significantly in HTR-8/SVneo and BeWo.

On the left, data are presented as mean±SD (n=3 biological replicates). Error bars are shown only in one direction for better readability. For some points, the error bars would be shorter than the height of the symbol and are not displayed. On the right, representative Western blots are shown. ^🞷^ p<0.05; ^🞷🞷^ p<0.01; ^🞷🞷🞷^ p<0.001.

**
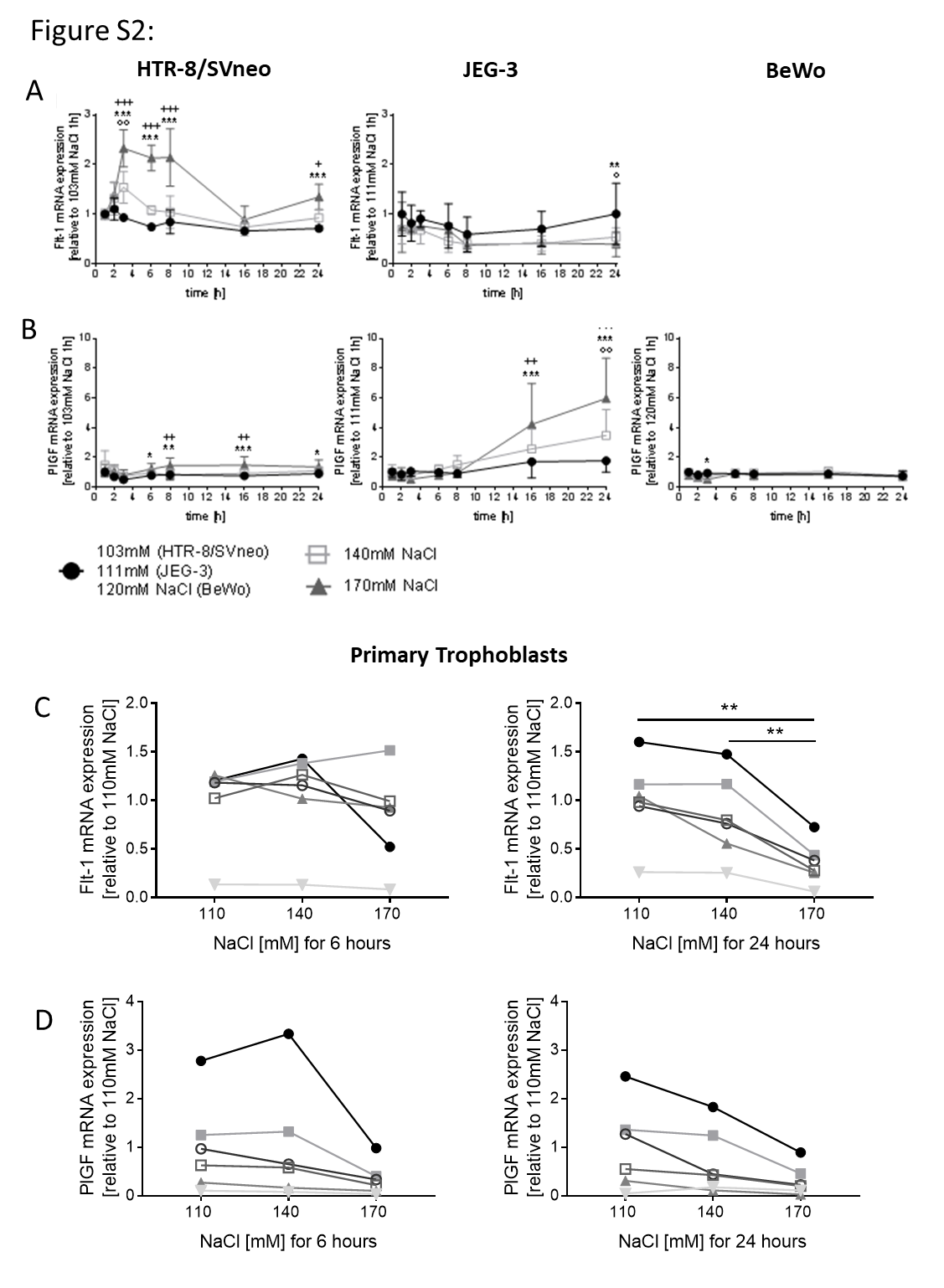
**

**Supplementary Figure S2:** Effect of different NaCl concentrations on mRNA expression of Flt-1 and PlGF in human trophoblast cell lines (A & B) and isolated primary term cytotrophoblasts (C & D). The human trophoblast cell lines HTR-8/SVneo (first trimester trophoblasts), JEG-3 and BeWo (trophoblasts, derived from choriocarcinoma cell lines), were incubated with normal (103 mM for HTR-8/SVneo, 111 mM for JEG-3, 120 mM for BeWo) or high NaCl concentrations (140 mM, 170 mM) for 1-24 hours. The mRNA expression of Flt-1 and PlGF was measured by qPCR. A&C) Flt-1 mRNA decreased after 24 hrs high NaCl in JEG-3 and primary CTBs, whereas HTR-8/SVneo showed an increase after 3-8hrs. B&D) High NaCl increased PlGF mRNA in JEG-3 and HTR-8/SVneo, yet not in BeWo and primary CTBs. A&B: Data are presented as mean ± SD (n=3 biological replicates). Error bars are shown only in one direction for better readability. For some points, the error bars would be shorter than the height of the symbol and are not displayed. ^🞷, 🞅^ or ^🞣^ *P*<0.05; ^🞷🞷, 🞅🞅 or 🞣🞣^ *P*<0.01; ^🞷🞷🞷, 🞅🞅🞅^ or ^🞣🞣🞣^ *P*<0.001. ^🞷^ shows significances between 170 mM NaCl and normal NaCl (103 mM for HTR-8/SVneo, 111 mM for JEG-3, 120 mM for BeWo). ^🞅^ shows significances between 140 mM NaCl and normal NaCl. ^🞣^ shows significances between 170 mM and 140 mM NaCl. C&D: Each symbol and shade of grey represents a different isolation of CTBs from an individual placenta (n=6). ^🞷^ *P*<0.05; ^🞷🞷^ *P*<0.01; ^🞷🞷🞷^ *P*<0.001.


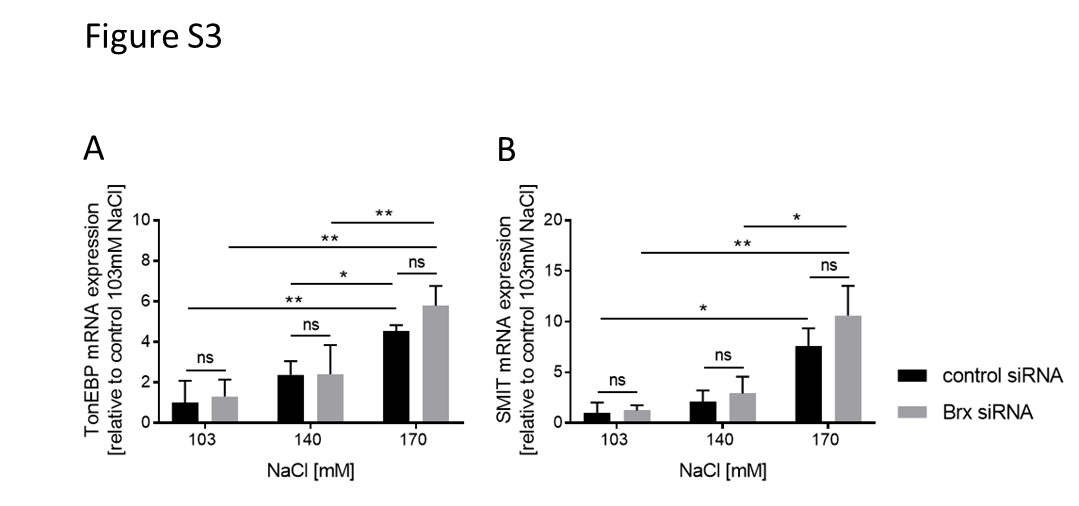


**Supplementary Figure S3:** Effect of Brx knockdown on mRNA expression of TonEBP and SMIT. Brx was knocked down in HTR-8/SVneo by means of siRNA and cells were incubated in medium with low (103 nM), normal (140 mM) or high (170 mM) NaCl. The mRNA expression of TonEBP (A) and SMIT (B) was measured by qRT-PCR. A 90% knockdown of Brx (data not shown) did not prevent the TonEBP or SMIT increase at high NaCl concentrations.
